# Harnessing Work Function Modulation for Hydrogen Evolution Catalysis in Mesoporous Bimetallic Pt‐M Alloys: The Role of Mesopores in Work Function Optimization

**DOI:** 10.1002/advs.202505464

**Published:** 2025-05-23

**Authors:** Lei Fu, Yunqing Kang, Ho Ngoc Nam, Kaiteng Wang, Zilin Zhou, Yingji Zhao, Quan Manh Phung, Kai Wu, Yusuke Asakura, Jun Zhou, Yusuke Yamauchi

**Affiliations:** ^1^ Center of Nanomaterials for Renewable Energy, State Key Laboratory of Electrical Insulation and Power Equipment Xi'an Jiaotong University Xi'an 710049 P. R. China; ^2^ Department of Materials Process Engineering, Graduate School of Engineering Nagoya University Nagoya 464‐8603 Japan; ^3^ Department of Chemistry, Graduate School of Science Nagoya University Furo‐cho, Chikusa‐ku Nagoya 464‐8602 Japan; ^4^ Institute of Transformative Bio‐Molecules (WPI‐ITbM) Nagoya University Furo‐cho, Chikusa‐ku Nagoya 464‐8601 Japan; ^5^ Australian Institute for Bioengineering and Nanotechnology (AIBN) The University of Queensland Brisbane QLD 4072 Australia; ^6^ Department of Convergent Biotechnology & Advanced Materials Science Kyung Hee University 1732 Deogyeong‐daero, Giheung‐gu Yongin‐si Gyeonggi‐do 17104 South Korea

**Keywords:** electrocatalysis, hydrogen evolution reaction, mesoporous film, mesoporous metallic alloy, work function

## Abstract

Work function (WF) influences electron transport and intermediates adsorption, enabling charge balance and catalytic optimization for the hydrogen evolution reaction (HER). However, the understanding of the role of mesopores and the relationship between composition and WF in pristine Pt‐based alloys remains lacking. Herein, various mesoporous binary Pt‐M alloy films (m‐Pt‐M, M = Pd, Rh, and Ru) with uniform pores and elemental distributions are synthesized, providing an experimental platform to investigate this relationship. It has been demonstrated that the WFs of m‐Pt‐M catalysts are strongly influenced by their compositions and mesoporous structures, thereby impacting HER activities. Among them, m‐Pt‐Ru with tailored WF lowers the thermodynamic energy barrier and accelerates the kinetic processes of HER. The mass activity of m‐Pt‐Ru in alkaline media is 17.8× and 5.1× higher, compared to Pt black and m‐Pt, respectively. This work not only provides a simple method for the fabrication of well‐defined binary metallic alloy films but also offers experimental insights into the rational design of highly efficient electrocatalysts with tunable WFs.

## Graphical abstract

This study presents mesoporous binary Pt‐M alloy films (m‐Pt‐M, M = Pd, Rh, and Ru) with uniform porosity and tailored work functions (WFs) as an experimental study platform. The WFs of the m‐Pt‐M films exhibit a volcano‐type relationship with their HER performance. Among them, the m‐Pt‐Ru demonstrates superior activity, requiring only 16 mV overpotential to reach 10 mA cm^−2^ in alkaline media.

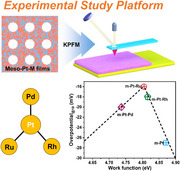

## 1 Introduction

Hydrogen plays an indispensable role in energy conversion and storage due to its zero‐carbon characteristic and high energy density.^[^ ^1^ ^]^ Notably, renewable electricity‐driven water electrolysis is regarded as one of the most promising approaches for hydrogen production.^[^ ^2^ ^]^ Despite recent advancements in catalyst development, Pt‐based catalysts remain the state‐of‐the‐art choice for the cathodic hydrogen evolution reaction (HER) in water electrolysis.^[^ ^3^ ^]^ It is crucial to provide experimental and theoretical guidance for the rational design of Pt‐based HER catalysts.

Taking Pt‐based alloys as an example, incorporating foreign metals into Pt optimizes metal‐hydrogen bond strength through electronic and/or geometric effects, enhancing electrocatalytic HER performance in accordance with the Sabatier principle.^[^ ^4^ ^]^ Significant efforts have been devoted to developing activity descriptors, such as *d*‐band center, adsorption energy, and occupancy of e_g_‐symmetry electrons, to describe and predict the catalytic performance of metal alloys.^[^ ^5^ ^]^ However, most studies are case‐by‐case and primarily emphasize theoretical predictions, with fewer efforts directed toward experimental strategies for catalyst design. Work function (WF) is a key surface property of materials, which represents the minimum energy needed to extract an electron from the Fermi level to the vacuum level.^[^ ^6^ ^]^ Modulating the WF of supported metal catalysts influences charge transportation, thereby altering the adsorption properties of hydrogen.^[^ ^7^ ^]^ Studying the effect of compositions on the WF requires eliminating the influence of other structural factors, such as metal size, support, morphology, and crystal phase. The reason lies in the substantial variations in the structures and compositional inhomogeneities of the catalysts, which hinder accurate evaluation of their intrinsic relationships. Thus, constructing controllable homogeneous structures to explore the structure‐performance relationships is essential for offering insights into the rational design of highly efficient catalysts. Although the remarkable achievements in mesoporous metal alloys developed by our group and beyond,^[^ ^8^ ^]^ rare studies are reported on the relationship between WF and compositions or porous structures in pristine Pt‐M alloys and their impact on HER performance.

Herein, various binary Pt‐M (M = Pd, Rh, and Ru) alloy mesoporous films with well‐defined pore sizes and uniform compositions are synthesized via a soft‐template‐assisted electrodeposition strategy. The WFs of these mesoporous Pt‐M alloy films (m‐Pt‐M), measured by Kelvin probe force microscopy (KPFM), exhibit a volcano‐type relationship with their HER activities and intrinsic kinetics. Among them, the m‐Pt‐Ru with an appropriate WF of 4.805 eV exhibits enhanced HER activity with a low overpotential of 16 mV to achieve a current density of 10 mA cm^−2^ in alkaline media. Experimental and theoretical calculations reveal that the appropriate WF of m‐Pt‐Ru makes it more suitable for electron transfer and reaction pathways in the HER, thereby improving catalytic performance. This strategy provides a high‐quality experimental platform for guiding the rational design of efficient catalysts and understanding the structure‐performance relationships.

## 2 Results and Discussion

### 2.1 Morphological and Structural Characterization

**Figure 1:**
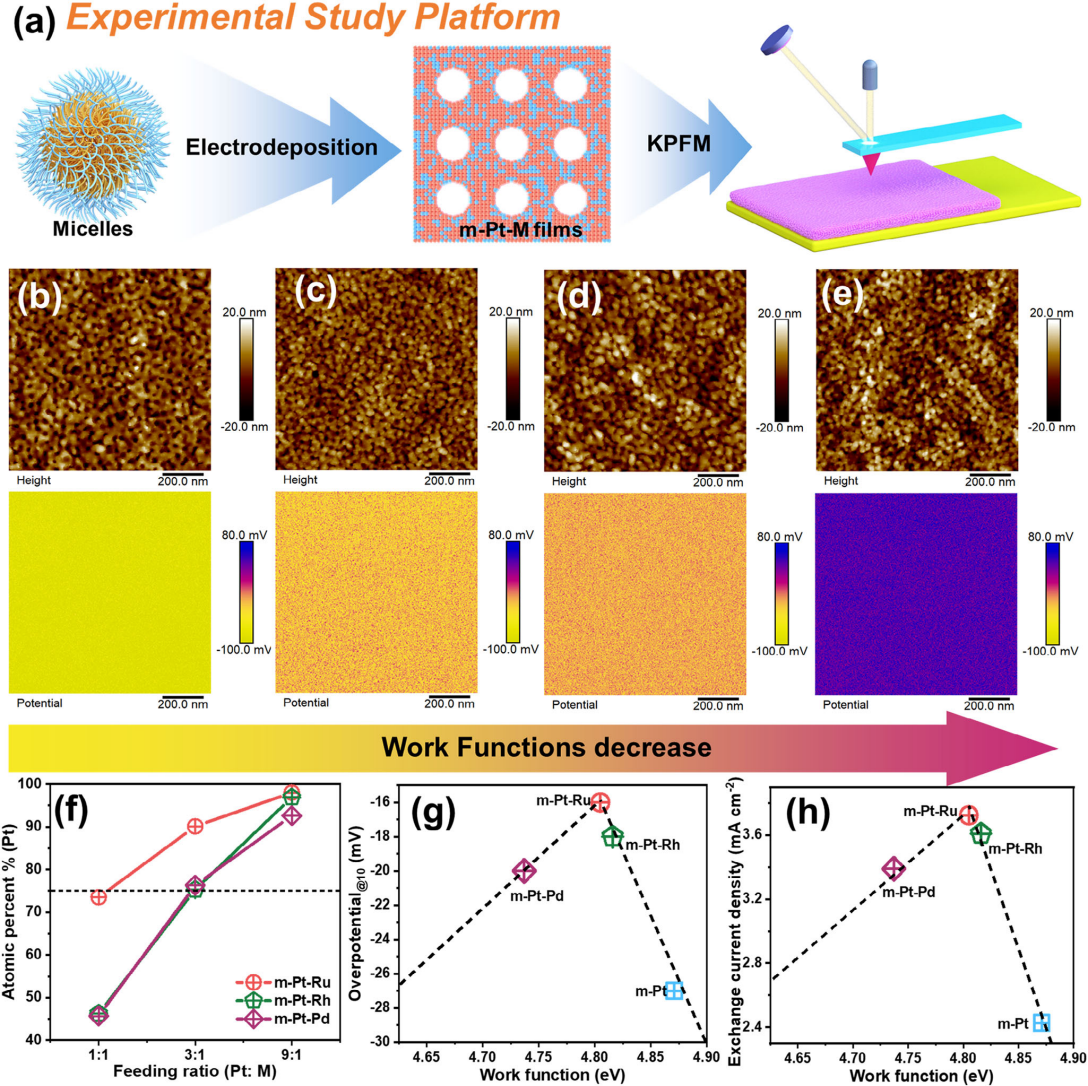
a) Schematic illustration of the experimental study platform for m‐Pt‐M. b–e) Height images (upper) and potential images (down) of (b) m‐Pt, (c) m‐Pt‐Rh (with an atomic ratio of approximately Pt:Rh = 3:1), (d) m‐Pt‐Ru (with an atomic ratio of approximately Pt:Ru = 3:1), and (e) m‐Pt‐Pd (with an atomic ratio of approximately Pt:Pd = 3:1). f) Atomic percentage of Pt in m‐Pt‐M with different feeding ratios, obtained from SEM‐EDS results. g) Correlation of HER overpotentials of m‐Pt and m‐Pt‐M (with a similar atomic ratio of approximately Pt:M = 3:1) and h) exchange current densities with their WFs.

Mesoporous Pt‐M (M = Rh, Ru, and Pd) alloy films were fabricated using a soft‐template‐assisted electrodeposition strategy (**Figure** **1**a). A cleaned Au‐coated Ti‐Si substrate was employed as a working electrode for electrodeposition in a micelle solution containing polystyrene‐*b*‐poly(oxyethylene) (PS_18000_‐*b*‐PEO_7500_) and metal ions (Figure S1, Supporting Information). Driven by a constant potential, these micelles decorated metal ions migrated to the working electrode, where the metal species was electrochemically reduced and gradually grew into a film.^[^ ^9^ ^]^ Subsequently, the soft template (i.e., PS_18000_‐*b*‐PEO_7500_ micelle) was removed, resulting in the formation of mesoporous films.

Scanning electron microscopy (SEM) images show m‐Pt, m‐Pt‐Pd, m‐Pt‐Rh, and m‐Pt‐Ru films exhibit highly distinct mesoporous structures with uniform pore size (Figures S2–S7, Supporting Information). All the films prepared using block copolymer PS_18000_‐*b*‐PEO_7500_ exhibit identical pore sizes (≈26 nm) as m‐Pt‐Ru and m‐Pt films (Figures S2c and S4d and S7, Supporting Information).

Additionally, energy dispersive spectroscopy (EDS) images confirm the uniform distribution of the corresponding elements, and the atomic ratio obtained from EDS is consistent with the result from inductively coupled plasma‐optical emission spectrometry (ICP‐OES, Table S1, Supporting Information). As shown in Figure 1f, the final compositional ratios of the m‐Pt‐M films can be controlled by adjusting the feeding ratios. To eliminate the effect of composition, the final Pt:M atomic ratio should be the same, as indicated by the dotted line (Figure 1f). Therefore, three m‐Pt‐M films with a similar atomic ratio of approximately Pt:M = 3:1 were selected as representative samples for this study. X‐ray diffraction (XRD) patterns for m‐Pt‐M films with a similar atomic ratio of approximately Pt:M = 3:1 reveal diffraction peaks corresponding to the face‐centered cubic (*fcc*) structure of Pt (No. 04–0802) with no impurity phases, indicating the successful formation of alloy (Figure S8, Supporting Information). Based on the above results, all the catalysts exhibit nearly identical structural features, including mesoporous structures and crystal phases. This makes it possible to directly compare their electrocatalytic performance by considering only the chemical compositions of the pristine binary metal films.

The electrochemical performance of these mesoporous films toward HER was evaluated in an N_2_‐saturated 1 M KOH solution. As shown in Figure S9 (Supporting Information), the linear sweep voltammetry (LSV) curves indicate an activity trend of m‐Pt‐Ru > m‐Pt‐Rh > m‐Pt‐Pd > m‐Pt, which highlights the various catalytic behavior of Pt group metals when alloyed with Pt. Furthermore, the Tafel slopes of m‐Pt‐M films are 37.7, 38.1, 44.0, and 42.3 mV dec^−1^ for m‐Pt‐Ru, m‐Pt‐Rh, m‐Pt, and m‐Pt‐Pd films, respectively, which is aligned with the observed overpotential trend. These Tafel values suggest that the Volmer–Heyrovsky mechanism is likely at work in alkaline media.^[^ ^10^ ^]^ Additionally, the observed variation in HER activity motivates further investigation into their structure‐performance relationship.

WF is a characteristic electronic property of catalysts that can be used to correlate with their catalytic performance.^[^ ^11^ ^]^ As a proof‐of‐concept study, KPFM was employed to analyze the variation in the WFs of prepared mesoporous films.^[^ ^12^ ^]^ The detailed principle of KPFM is similar to that described in our previous publications.^[^ ^13^ ^]^ Initially, the WF of the conductive probe (SCM‐PIT‐V2) was calibrated using highly oriented pyrolytic graphite, owing to its well‐known WF of 4.60 eV (Figure S10, Supporting Information).^[^ ^14^ ^]^ In Figure 1b–e, the topography images confirm that all m‐Pt‐M films with a similar atomic ratio of approximately Pt:M = 3:1 maintain their mesoporous structure. It is noted that the pore sizes observed in topography images appear smaller than those in SEM images due to the tip size effect.^[^ ^15^ ^]^ However, the contact potential difference (CPD) was captured in the lift mode with the lift height of 50 nm, minimizing the influence of tip size and sample topography.

A series of m‐Pt‐Ru films (with an atomic ratio of approximately Pt:Ru = 3:1) with varying thicknesses was synthesized under identical conditions by adjusting the deposition time (Figure S11, Supporting Information). Cross‐sectional SEM images reveal that the thickness of the m‐Pt‐Ru films increases linearly with deposition time at a growth rate of 0.166 nm s^−1^ (Figure S11f, Supporting Information). The WFs of the m‐Pt‐Ru films with different thicknesses exhibit an initial increase, followed by a plateau, indicating that the WF remains almost unchanged after reaching a certain thickness (Figure S12, Supporting Information). Moreover, this trend is also consistent with the observed HER performance, thus the optimal m‐Pt‐Ru film is selected with a deposition time of 1200 s (Figure S13, Supporting Information).

To investigate the role of mesopores, nonporous Pt‐Ru and m‐Pt‐Ru with different sizes of mesopores were also synthesized (Figures S4 and S14a–c, Supporting Information). LSV curves show that the HER performance of m‐Pt‐Ru films is superior to that of the nonporous film, confirming that the mesopores are favorable for the enhancement of catalytic activity (Figure S14d, Supporting Information). Additionally, a clear difference in the work function of the nonporous and mesoporous Pt‐Ru films further indicates that the mesopores have a significant effect on the WF (Figure S14e, Supporting Information). This is attributed to mesopore‐induced charge redistribution, where the degree of curvature influences surface dipole interactions, resulting in variations in the electric field, consistent with recently reported literature.^[^ ^16^ ^]^ Among the three types of mesopore sizes (18, 26, and 41 nm; Figure S4, Supporting Information), the m‐Pt‐Ru film with an intermediate pore size of 26 nm demonstrates superior electron transport capabilities, as also evidenced by the reduced charge transfer resistance in electrochemical impedance spectroscopy (EIS) measurements (Figure S15 and Table S2, Supporting Information). These 26 nm‐pore m‐Pt‐M configurations were subsequently employed to systematically investigate the compositional influence on WF modulation. The measured WF of m‐Pt film is 4.871 eV, consistent with previously reported values.^[^ ^17^ ^]^ Note that the experimentally measured WF is slightly different from the theoretical values, which is caused by the fact that the KPFM is carried out under an air atmosphere. The WFs of m‐Pt‐M films were determined to follow this trend: m‐Pt (4.871 eV) > m‐Pt‐Rh (4.816 eV) > m‐Pt‐Ru (4.805 eV) > m‐Pt‐Pd (4.737 eV). These results suggest that the incorporation of heteroatoms reduces the WFs of the m‐Pt‐M films. Subsequently, the relationship between HER activity and WFs was plotted. Figure 1g shows that the alkaline HER performance exhibits a volcano‐type relationship with the WFs. Notably, the m‐Pt‐Ru film, formed by alloying Pt with Ru, is positioned at the top of the volcano plot, indicating the highest catalytic activity among the films. Additionally, EIS measurements reveal that the m‐Pt‐Ru film exhibits the lowest charge transfer resistance among m‐Pt and other m‐Pt‐M films, reflecting accelerated electron transfer kinetics (Figure S16 and Table S3, Supporting Information).

Exchange current density is another indicator for assessing the charge transfer between catalysts and intermediates.^[^ ^18^ ^]^ The m‐Pt‐Ru film, featuring a high exchange current density, suggests an optimal barrier for charge transfer and intermediate binding strength (Figure 1h). To further validate the volcano‐type relationship between HER activity and WF, m‐Pt‐Ir and m‐Pt‐Au films were synthesized, structurally characterized, and evaluated for their HER activity (Figure S17, Supporting Information). The m‐Pt‐M films prepared with different feeding ratios were also measured (Figures S18–S20, Supporting Information) and plotted in Figure S21 (Supporting Information). The results show that m‐Pt‐Rh films are positioned on the right side of the volcano‐type map with relatively high WFs, while m‐Pt‐Pd films are located on the left side with low WFs. Interestingly, m‐Pt‐Ru films with optimized WFs are positioned at the peak of the volcano plot. Moreover, it is noted that the trends in exchange current density and Tafel slope as functions of WFs align well with the expected correlation. These results demonstrate that tuning the WF is a valuable strategy for understanding the structure‐performance relationships and rational design of catalysts.

**Figure 2:**
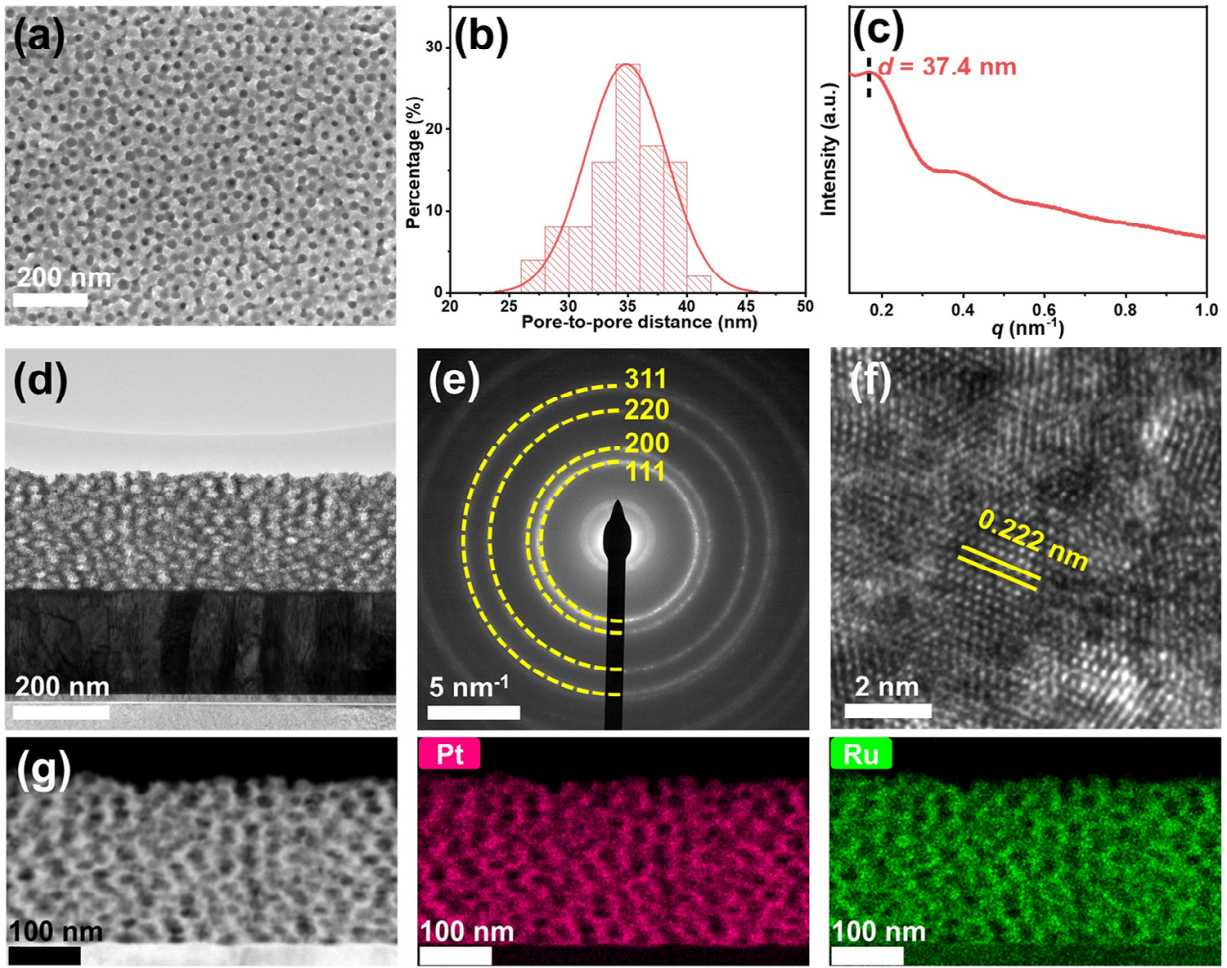
a) Top‐view SEM image, b) pore‐to‐pore distance, c) SAXS pattern, d) TEM image, e) SAED pattern, f) HRTEM image, g) HAADF image, and corresponding EDS maps of m‐Pt‐Ru film.

Typically, we systematically investigated the microstructure and electronic properties of the optimum m‐Pt‐Ru film. Top‐view SEM image shows that the surface of the m‐Pt‐Ru film contains a substantial amount of uniformly sized mesopores with an average diameter of 26 nm, consistent with the size of the spherical micelles reported previously (**Figure** **2**a).^[^ ^19^ ^]^ The pore‐to‐pore distance obtained from the SEM image is 35 nm (Figure 2b). Additionally, small‐angle X‐ray scattering (SAXS) pattern displays an intense peak located at *q* = 0.168 nm^−1^ for m‐Pt‐Ru film (Figure 2c), corresponding to a pore‐to‐pore distance of 37.4 nm, in agreement with the result from the SEM image. The SAXS pattern also suggests that m‐Pt‐Ru possesses localized ordering in its pore structure, as corroborated by the SEM image. Moreover, the cross‐sectional structure of the m‐Pt‐Ru film, captured using a focused ion beam and transmission electron microscopy (TEM), reveals a well‐defined mesoporous architecture throughout the entire film (Figure 2d). The selected area electron diffraction (SAED) pattern confirms the single *fcc* structure of the Pt‐Ru alloy (Figure 2e), aligning well with the XRD results (Figure S8, Supporting Information). High‐resolution TEM (HRTEM) image indicates the polycrystalline nature of the film, with a lattice fringe of 0.222 nm attributed to the (111) crystal plane of the m‐Pt‐Ru film (Figure 2f). Compared to the (111) plane spacing of bulk Pt (0.226 nm),^[^ ^20^ ^]^ the smaller lattice distance in the m‐Pt‐Ru film suggests compressive strain, which can modify the surface electronic structure, thereby enhancing electrochemical activity. High‐angle annular dark‐field (HADDF) image and EDS elemental mapping reveal a homogeneous distribution of Pt and Ru elements in the m‐Pt‐Ru film (Figure 2g). Additionally, the uniform composition across different regions of the film, as evidenced by EDS mapping (Figure S22, Supporting Information), highlights the homogeneity of the mesoporous films. We emphasize that the m‐Pt‐Ru film not only provides a homogeneous composition distribution and a well‐defined mesoporous structure, but also features a high electrochemically active surface area and a modified electronic structure, both of which are advantageous for enhancing catalytic activities.

**Figure 3:**
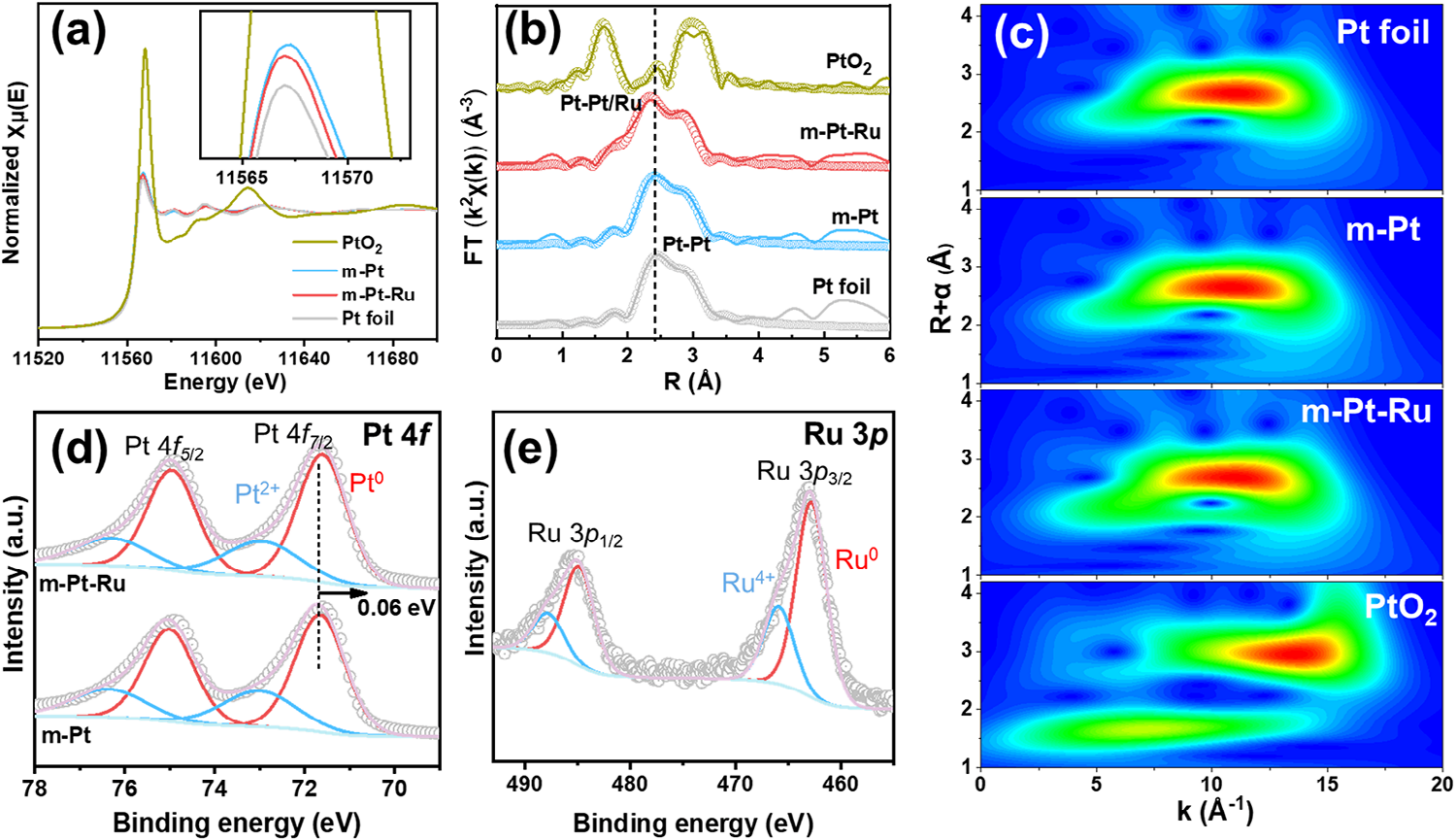
a) Normalized Pt L_3_‐edge XANES spectra of m‐Pt, m‐Pt‐Ru, Pt foil, and PtO_2_. b) Pt L_3_‐edge EXAFS spectra and corresponding fitting curves of m‐Pt, m‐Pt‐Ru, Pt foil, and PtO_2_. c) Wavelet transformed EXAFS spectra of m‐Pt, m‐Pt‐Ru, Pt foil, and PtO_2_. d) Pt 4*f* XPS spectra of m‐Pt and m‐Pt‐Ru. e) Ru 3*p* XPS spectrum of m‐Pt‐Ru.

Consequently, we employed X‐ray absorption near‐edge spectroscopy (XANES) and extended X‐ray absorption fine structure (EXAFS) to further analyze the valence state and coordination structure of Pt species. **Figure** **3**a shows the normalized XANES spectra of the Pt L_3_‐edge for the m‐Pt‐Ru and m‐Pt, as well as the referenced Pt foil and PtO_2_. The intensities of white‐line peaks for both the m‐Pt and m‐Pt‐Ru are close to that of Pt foil but significantly lower than that of PtO_2_, suggesting that Pt primarily exists in the metallic state.^[^ ^21^ ^]^ Additionally, the white‐line peak intensity of m‐Pt‐Ru is lower than that of m‐Pt, suggesting charge transfer from Ru to Pt due to differences in electronegativity.^[^ ^22^ ^]^ This could result in the electron enrichment around Pt atoms, which favors the enhanced HER activity. The EXAFS spectra were further analyzed to elucidate the coordination structures of mesoporous films (Figure S23, Supporting Information). Compared to Pt foil, the mesoporous films show similar features in their EXAFS spectra, further confirming the metallic state of Pt, with the main peak assigned to Pt‐Pt/Ru bonds (Figure 3b). Notably, the Pt−Pt/Ru bond distance in the m‐Pt‐Ru is slightly shorter than that in the m‐Pt and Pt foil, which can be attributed to the formation of a Pt‐Ru alloy with smaller lattice parameters and compressive strain, consistent with the HRTEM result. The wavelet transforms (WT) of the EXAFS spectra also confirm the difference in the coordination structure of Pt‐Pt/Ru bonds between the m‐Pt‐Ru and m‐Pt films (Figure 3c). In addition, the surface chemical valence states of the mesoporous films were examined using X‐ray photoelectron spectroscopy (XPS). The Pt 4*f* XPS spectra are deconvoluted into metallic Pt^0^ and Pt^2+^ species (Figure 3d). And the Ru 3*p* spectrum of the m‐Pt‐Ru is designated to Ru^0^ and Ru^4+^ (Figure 3e). It is evident that both Pt and Ru are predominantly in the metallic state. Notably, the binding energy of Pt in m‐Pt‐Ru is shifted negatively by 0.06 eV compared to m‐Pt, further confirming the charge transfer effect in m‐Pt‐Ru, consistent with the XAFS results.

### 2.2 HER Electrocatalytic Performance

**Figure 4:**
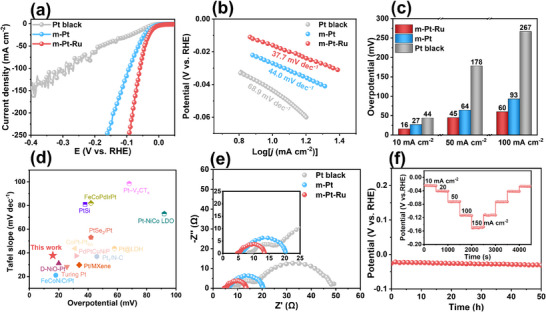
a) LSV curves, b) Tafel slopes, and c) overpotentials at different current densities of m‐Pt, m‐Pt‐Ru, and commercial Pt black in 1 M KOH electrolyte. d) Comparison of HER performance for m‐PtRu film with recently reported Pt‐based catalysts in alkaline solution.^[^ ^23^ ^]^ e) EIS Nyquist plots and fitting curves of m‐Pt, m‐Pt‐Ru, and commercial Pt black. f) Chronopotentiometry test of m‐Pt‐Ru at a current density of 10 mA cm^−2^. The insert is a multi‐step chronopotentiometry test of m‐Pt‐Ru.

The HER electrocatalytic performance of the m‐Pt‐Ru, m‐Pt, and commercial Pt black was systematically evaluated in N_2_‐saturated 1 M KOH electrolyte. As shown in **Figure** **4**a, the m‐Pt‐Ru film exhibits the best HER electrocatalytic performance with a low overpotential of 16 mV at a current density of 10 mA cm^−2^, superior to that of the m‐Pt film (27 mV) and commercial Pt black (44 mV). This performance is also comparable to recently reported Pt‐based catalysts (Figure 4d; Table S4, Supporting Information).^[^ ^23^ ^]^ Additionally, the m‐Pt‐Ru shows the lowest Tafel slope of 37.7 mV dec^−1^, indicating enhanced intrinsic activity (Figure 4b).^[^ ^10^ ^]^ The mass activity of the m‐Pt‐Ru at an overpotential of 100 mV is 17.8 and 5.1 times higher than that of Pt black and m‐Pt film, respectively (Figure S24, Supporting Information). Furthermore, the m‐Pt‐Ru requires a low overpotential of 60 mV to deliver a high current density of 100 mA cm^−2^, highlighting its potential as an efficient electrocatalyst for practical water electrolysis (Figure 4c). Moreover, the double‐layer capacitance (*C*_dl_) was assessed via cyclic voltammetry at various scan rates to evaluate the intrinsic activity of the catalysts (Figure S25, Supporting Information). Since the catalyst is without carbon support, *C*_dl_ has a positive correlation with the electrochemically active surface area (ECSA).^[^ ^23c^ ^]^ The m‐Pt‐Ru exhibits the highest *C*_dl_ value (72.3 mF cm^−2^), indicating a higher ECSA and more exposed active sites, which contribute to its superior HER catalytic activity. Furthermore, the turnover frequency (TOF) of the m‐Pt‐Ru surpasses that of both m‐Pt and Pt black, further confirming its enhanced intrinsic activity (Figure S26, Supporting Information).

Electrochemical impedance spectroscopy (EIS) was employed to investigate charge transfer capabilities. The lowest charge transfer resistance (*R*_ct_) observed for m‐Pt‐Ru provides strong evidence for its superior charge transfer kinetics (Figure 4e). In situ EIS measurements were conducted to further explore the origin of the improved HER kinetics. The Nyquist plots were fitted using a double‐parallel equivalent circuit model, where the first parallel component reflects the charge transfer process, and the second captures hydrogen adsorption behavior (Figure S27, Supporting Information).^[^ ^24^ ^]^ The pseudo‐capacitance (C1) at different potentials was plotted to track the hydrogen adsorption behavior during the HER process (Figure S28, Supporting Information). Commercial Pt black exhibits a small, potential‐independent C1 value, whereas the m‐Pt‐Ru film exhibits potential‐dependent behavior. Considering that alkaline HER involves water adsorption‐dissociation and hydrogen adsorption‐dissociation processes, the C1 could be categorized into two regions. At low overpotentials, the lower C1 values of the m‐Pt‐Ru compared to the m‐Pt suggest weakened hydrogen adsorption and increased water adsorption site availability. At high overpotentials, the C1 values of the m‐Pt‐Ru increase significantly with potential, indicating enhanced hydrogen adsorption and coverage, which favor the subsequent H_2_ formation step. It is worth mentioning that Pt is known to have a stronger hydrogen binding strength, while Ru reduces the water dissociation barrier.^[^ ^25^ ^]^ Therefore, the alloyed m‐Pt‐Ru is expected to yield an optimal bonding strength for intermediates, induced by Ru modification, which is responsible for its excellent alkaline HER performance.

Chronopotentiometry tests were conducted to evaluate the catalytic stability of the m‐Pt‐Ru. As shown in Figure 4f, the potential of the m‐Pt‐Ru responds rapidly and remains stable across different current densities (from 10 to 150 mA cm^−2^), indicating efficient mass transport and exceptional durability. Post‐reaction characterizations, including SEM, TEM, SAED, HRTEM, XRD, and EDS mapping, reveal negligible changes in the mesoporous morphology, crystal structure, and elemental compositions (Figures S29 and S30, Supporting Information). Besides, the XPS spectra of Pt 4*f* and Ru 3*p* show a slight negative shift after the stability test, suggesting electrochemical reduction of surface metal elements during the HER process (Figure S31, Supporting Information). Given the instability of Si substrate in alkaline solutions, the m‐Pt‐Ru was electrochemically deposited on carbon paper to assess long‐term stability (Figure S32, Supporting Information). The mesoporous morphology, composition, and HER activity are similar to those observed on the Au‐Ti‐Si substrate (Figure S33, Supporting Information). Chronopotentiometry curves demonstrate that the m‐Pt‐Ru maintained more stable performance with a slight potential increase of only 5 mV over a 50‐h continuous test compared to m‐Pt‐Rh and m‐Pt‐Pd films (Figure 4f; Figure S34, Supporting Information). The mesopore and composition of the m‐Pt‐Ru were well‐preserved after the stability test, further confirming its excellent HER stability (Figure S35, Supporting Information). Moreover, the Faraday efficiency of hydrogen production was determined to be 99.6%, implying the electrons were mainly employed for hydrogen generation without side reactions (Figure S36, Supporting Information). Overall, the m‐Pt‐Ru exhibits high activity, cost‐effectiveness, and excellent stability for alkaline HER, showcasing its promising potential for practical applications.

**Figure 5:**
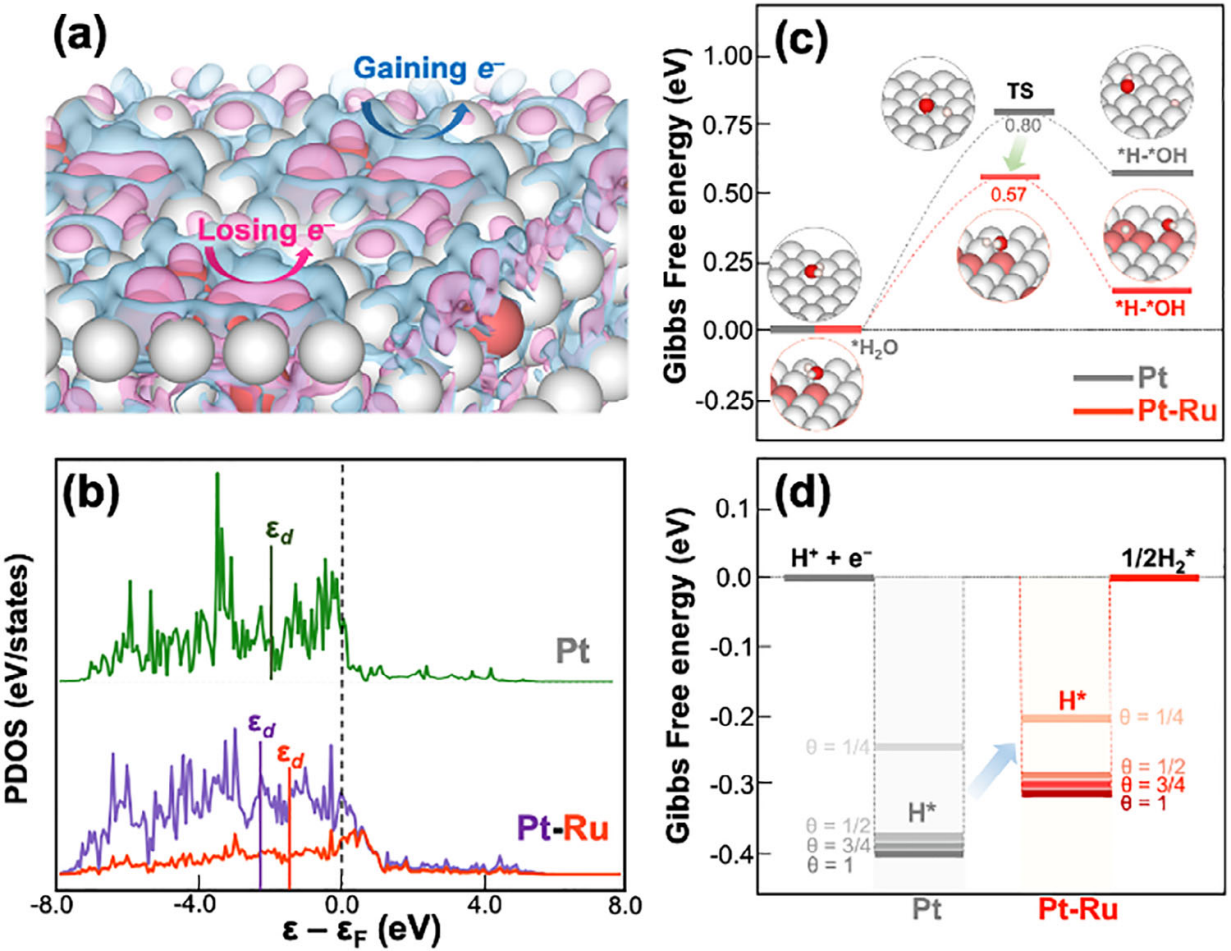
a) Charge density distribution on Pt‐Ru surface (cyan: gaining electron region and pink: losing electron region, isosurface value of 0.001 e.Bohr^−3^). b) PDOSs and their corresponding *d*‐band centers of single Pt and Pt‐Ru systems. Gibbs free energy of c) water dissociation and d) hydrogen adsorption at different coverage ratios on single Pt and Pt‐Ru surfaces for the HER process.

Density functional theory (DFT) calculations were conducted to elucidate the enhanced electrocatalytic performance of the bimetallic Pt‐Ru system compared to single Pt system (**Figure** **5**a–d). As shown in Figure 5a, introducing different atoms (e.g., Ru) led to charge density redistribution around Pt atoms. Specifically, due to the larger electronegativity of Pt, an enrichment of electron density was concentrated around Pt atoms, leaving deficient electron regions around their neighboring metal atoms (Table S5, Supporting Information). This redistribution modifies the electronic structures of the binary alloy system, potentially enhancing their HER performance. Indeed, the calculated partial density of states (PDOS) and its corresponding *d*‐band centers reveal the relation to the catalytic activity of these systems (Figure 5b). The downshift of the *d*‐band center of Pt within the Pt‐Ru system (**ε** *_d_* = −2.31 eV) places it farther from the Fermi level compared to that in a pure Pt system (**ε** *_d_* = −2.05 eV). This shift suggests fine‐tuning of the electronic structure, enabling adsorption without excessively strong binding to adsorbates, which is beneficial for improved catalytic activity.

To further understand the catalytic performance of Pt‐Ru, we estimated the HER process under alkaline conditions via two main reactions: the water dissociation steps (H_2_O + e^−^ + * → *H + OH^−^) and hydrogen adsorption (*H → 1/2H_2_). Figure 5c presents the adsorption Gibbs free energy of the water dissociation step. The dissociation of *H_2_O molecule into *H‐*OH occurs more readily on Pt‐Ru than on the Pt system. In particular, the activation energy for this reaction is 0.8 eV on the Pt surface, while it is significantly lower at ≈0.57 eV on the Pt‐Ru surface. According to the Evans–Polanyi principle, this reduction in activation energy is reasonably attributed to the stronger bonding of the *H‐*OH species on the Pt‐Ru surface. This reduction in the activation energy allows for improved water dissociation, directly contributing to optimizing HER activity on the binary alloy surfaces. The adsorption Gibbs free energy of H* on metallic surfaces with different coverage (θ) ratios was also calculated (Figure 5d). Accordingly, the pure Pt system has pretty ideal H adsorption free energies, which slightly vary depending on the surface coverage ratio as −0.24, −0.37, −0.39, and −0.40 eV at θ = 1/4, 1/2, 3/4, and 1, respectively. For the binary Pt‐Ru alloy, H absorption free energy is even further optimized, which is −0.20, −0.29, −0.31, and −0.32 eV at θ = 1/4, 1/2, 3/4, and 1, respectively. The minimization in adsorption strength of H* suggests a well‐balanced interaction between adsorption and desorption on the Pt‐Ru surface, facilitating the HER activity more efficiently than the Pt system. The above experimental and theoretical observations collectively demonstrate the superior performance of the binary m‐Pt‐Ru alloy film for efficient HER activity.

## 3 Conclusion

In summary, we have successfully synthesized a series of m‐Pt‐M alloy films with well‐defined structures using a soft‐template‐assisted electrodeposition strategy. Through experimental and theoretical approaches, we reveal that the WFs of m‐Pt‐M are strongly affected by their compositions and mesoporous structures, thereby modulating their intrinsic kinetics and HER performance. Typically, the m‐Pt‐Ru with a tailored WF uncovered in this study demonstrates superior HER performance under alkaline conditions, achieving a low overpotential of 16 mV at 10 mA cm^−2^, outperforming commercial Pt black and other Pt‐based alloys. The mass activity of m‐Pt‐Ru at an overpotential of 100 mV is 17.8× and 5.1× higher than that of Pt black and m‐Pt, respectively. This study provides an experimental platform and theoretical guidance for exploring the relationship between the compositions and WFs of binary alloy films and their impact on HER performance. Moreover, it lays a foundation for future investigations into the correlation between more complex metal alloys, such as medium‐entropy and high‐entropy alloys, and their electrocatalytic performance.

## Conflict of Interest

The authors declare no conflict of interest.

## Supporting information



Supporting Information

## Data Availability

The data that support the findings of this study are available in the supplementary material of this article.
