# Supplementary material for: Harnessing Work Function Modulation for Hydrogen Evolution Catalysis in Mesoporous Bimetallic Pt‐M Alloys: The Role of Mesopores in Work Function Optimization
Source: Adv Sci (Weinh). 2025 May 23;12(30):e05464. doi: 10.1002/advs.202505464 (PMC12376568; doi:10.1002/advs.202505464)
Supplement: Supplementary file 1 — Supporting Information [file ADVS-12-e05464-s001.docx]

**Methods**

**Materials.**

All chemicals and solvents were used as received without further purification. The block copolymers polystyrene-b-poly(oxyethylene) (PS_10000_-b-PEO_4100_, PS_18000_-b-PEO_7500_, PS_63000_-b-PEO_26000_) with different molecular weight were procured from Polymer Source. Potassium tetrachloroplatinate(II) (K_2_PtCl_4_, 98%), sodium tetrachloropalladate(II) (Na_2_PdCl_4_, 98%), sodium hexachlororhodate(III) (Na_3_RhCl_6_), ruthenium(III) chloride hydrate (RuCl_3_·xH_2_O, 99.98% trace metals basis), gold(III) chloride trihydrate (HAuCl_4_·3H_2_O, ≥99.9% trace metals basis), iridium(III) chloride hydrate (IrCl_3_·xH_2_O, 99.9% trace metals basis), and Nafion™ perfluorinated resin solution (5 wt%) were sourced from Sigma Aldrich. Tetrahydrofuran (THF), hydrochloric acid (HCl, 2M), and potassium hydroxide (KOH) were obtained from Nacalai Tesque. Platinum black was acquired from Alfa Aesar. Milli-Q water (18.2 MΩ·cm) was used for all experiments.

**Preparation of m-Pt-M (M = Pd, Rh, Ru, Ir and Au) film.**

In a typical procedure, 4 mg of polystyrene-*b*-poly(oxyethylene) (PS_18000_-*b*-PEO_7500_) was dissolved in 0.6 mL of tetrahydrofuran (THF) *via* ultrasonication. Then 0.5 mL of ethanol, 2.32 mL of deionized water, and 0.08 mL of hydrochloric acid (2 M HCl) were added to form a micellar solution at 40 °C. Then total 0.5 mL aqueous precursor solution with appropriate ratios of K_2_PtCl_4_ (40 mM) and another metal salt solution (40 mM; Na_2_PdCl_4_, Na_3_RhCl_6_, RuCl_3_·*x*H_2_O, IrCl_3_·xH_2_O or HAuCl_4_·3H_2_O) were dropwise added to the above solution under continuous stirring. The obtained precursor solutions were used as an electrolyte for the typical electrodeposition of mesoporous films at a contact potential of -0.2 V (*vs*. Ag/AgCl) for 1200 s. A 20 mL voltammetry cell was employed as an electrochemical cell. A conventional three-electrode system was employed for electrodeposition, in which a Pt wire, an Ag/AgCl electrode (in 3 M KCl), and an Au-coated Si wafer (3 mm × 15 mm) were used as a counter, reference, and working electrode, respectively. After electrodeposition, the working electrode was taken out and immersed in THF for 12h at room temperature to remove the micelles.

**Characterization**

X-ray Diffraction (XRD) patterns were collected by a Rigaku SmartLab 3kW-BU diffractometer with a step of 0.02°. Scanning electron microscopy (SEM) was carried out using a Hitachi SU-8000 with an energy-dispersive X-ray spectroscopy (EDS) system (Bruker 5060F). The transmission electron microscopy (TEM) and high-resolution transmission electron microscopy (HRTEM) images were obtained on a Talos F200X equipped with a Super-X EDS system (Super-X G2). For cross-sectional TEM observation, a Focused Ion Beam system (NX5000) was employed for the preparation of TEM specimens with an accelerating voltage of 30 kV. X-ray photoelectron spectroscopy (XPS) measurements were conducted using PHI Quantes. Kelvin probe force microscopy (KPFM) measurement was performed on a Bruker Dimension Icon system. Inductively coupled plasma‒optical emission spectrometry (ICP-OES) was implemented using an Agilent 5800 system.

**Electrochemical measurement**

All electrochemical tests were performed in N_2_-saturated 1 M KOH electrolyte and maintained the flow of N_2_ throughout all the electrochemical measurements. A graphite rod, a Hg/HgO electrode, and a mesoporous film were utilized as a counter, a reference, and a working electrode, respectively. For comparison, platinum black (3 mg) was dispersed into 1 mL solution containing 980 μL of ethanol and 20 μL of 5 wt% Nafion, followed by ultrasonication for 30 min. Then, 20 μL of the above ink was drop-cast onto a glassy carbon rotating disk electrode. The reference electrode was calibrated to the reversible hydrogen electrode (RHE) with the calculation formula: *E*_RHE_ = *E*(Hg/HgO) + 0.059×pH+0.098 V. LSV curves were collected at a scan rate of 5 mV s^-1^ with a 95%-iR compensation. Double-layer capacitance (*C*_dl_) was evaluated from CV curves at different scan rates from 5 – 25 mV s^-1^. In-situ Electrochemical impedance spectroscopy (EIS) was recorded from 100 kHz to 0.05 Hz on a BioLogic potentiostat. The stability test was conducted using chronopotentiometry at different current densities.

**Computational methods**

The plane-wave DFT calculations were performed in the Vienna *Ab initio* Simulation Package^[1]^ (VASP) utilizing the projector-augmented wave (PAW) method^[2]^ with a cut-off energy of 450 eV, where H 1*s*^1^, O 2*s*^2^2*p*^4^, Pt 5*d*^9^6*s*^1^, and Ru 4*d*^7^5*s*^1^ were treated as valence states. For the exchange-correlation part, the GGA-PBE functional^[3]^ was employed along with Grimme’s van der Waals correction (D3-BJ)^[4]^ to handle the dispersion forces of substrates and adsorbates. The calculated slab models were constructed by a 4 × 4 unit cell. Except for atoms in two bottom layers fixed to mimic bulk nature, the remaining atoms in the systems were allowed to fully relax until the residual forces and energy conditions were below 3×10^−2^ eVÅ^−1^ and 10^-4^ eV, respectively. Brillouin zone was sampled within Γ-centered *k*-meshes of 4 × 4 × 1. A vacuum layer of 15 Å was inserted along the *z*-direction of slabs to eliminate the undesired Coulombic interactions caused by periodic boundary conditions.

The Gibbs free energy of adsorbates was determined using the following relation: *G* = *E* + *ZPE* ‒ *TS*, where *E* is the total energy of the system, *ZPE* is a zero-point energy correction, and *TS* is the vibrational entropy of the adsorbed intermediate^[5,6]^. The HER activity was then considered by their relative free energy (Δ*G* = Δ*E* + Δ*E*_ZPE_ ‒ *T*Δ*S*) at standard conditions (*p* =1 atm and *T* = 298.15 K). A computational hydrogen electrode model by Nørskov was employed in proton-electron transfer steps, i.e., *G*(H^+^ + e^–^) equals *G*(1/2H_2_(g)). For the water dissociation reaction, the energy barriers of transition states (TS) were estimated using the climbing image nudged elastic band (CINEB) method^[7]^.

**REFERENCES**

[1] Kresse, G.; Furthmüller, J. Efficiency of ab-initio total energy calculations for metals and semiconductors using a plane-wave basis set. Comput. Mater. Sci. **1996**, 6, 15-50. DOI: 10.1016/0927-0256(96)00008-0.

[2] Perdew, J. P.; Burke, K.; Ernzerhof, M. Generalized Gradient Approximation Made Simple. Phys. Rev. Lett. **1996**, 77, 3865. DOI: 10.1103/PhysRevLett.77.3865.

[3] Kresse, G.; Joubert, D. From ultrasoft pseudopotentials to the projector augmented-wave method. Phys. Rev. B **1999**, 59, 1758. DOI: 10.1103/PhysRevB.59.1758.

[4] Grimme, S.; Ehrlich, S.; Goerigk, L. Effect of the damping function in dispersion corrected density functional theory. J. Comput. Chem. **2011**, 32, 1456-1465. DOI: 10.1002/jcc.21759.

[5] Nam, H. N.; Phung, Q. M.; Choeichom, P.; Yamauchi, Y.; Saito, N. First-principles studies of enhanced oxygen reduction reactions on graphene-and nitrogen-doped graphene-coated platinum surfaces. *Phys. Chem. Chem. Phys.*, **2024**, *26*, 10711-10722. DOI: 10.1039/D4CP00269E.

[6] Wang, V.; Xu, N.; Liu, J.-C.; Tang, G.; Geng, W.-T. VASPKIT: A user-friendly interface facilitating high-throughput computing and analysis using VASP code. Comput. Phys. Commun. **2021**, 267, 108033. DOI: 10.1016/j.cpc.2021.108033.

[7] Nørskov, K. J.; Rossmeisl, J.; Logadottir, A.; Lindqvist, L.; Kitchin, J. R.; Bligaard, T.; Jónsson, H. Origin of the Overpotential for Oxygen Reduction at a Fuel-Cell Cathode. *J. Phys. Chem. B* **2004**, *108*, 17886–17892. DOI: 10.1021/jp047349j


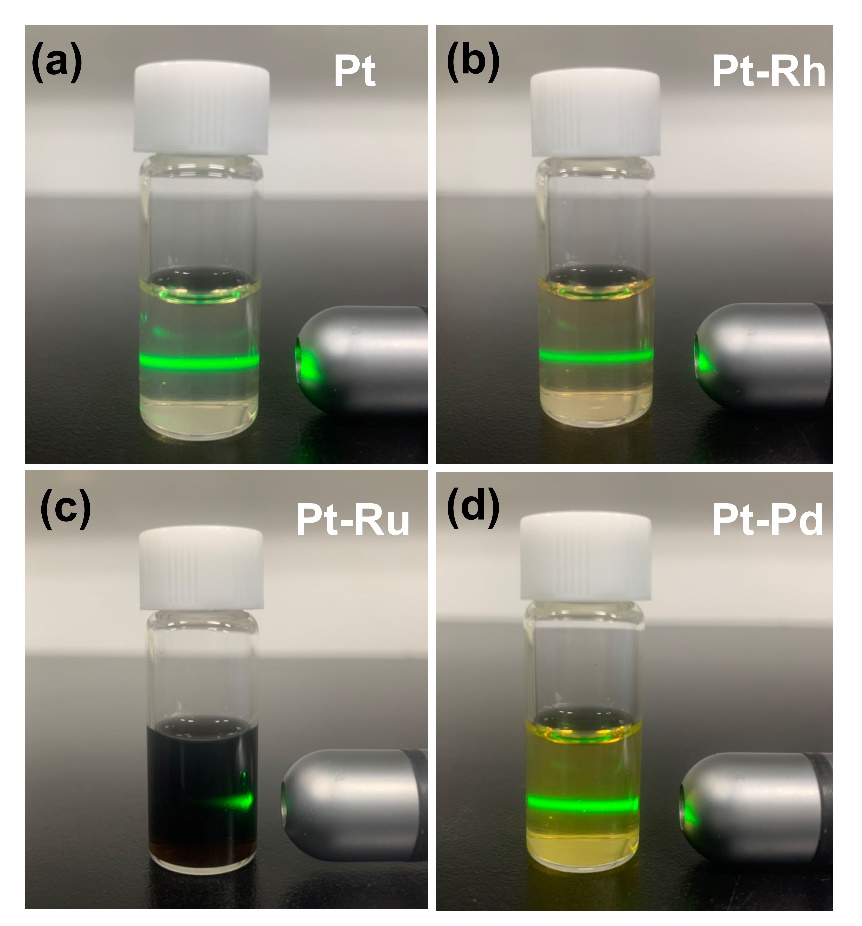


**Figure S1.** Photographs demonstrating the Tyndall effect in solution with different compositions. (a) K_2_PtCl_4_, (b) K_2_PtCl_4_ + Na_3_RhCl_6_, (c) K_2_PtCl_4_ + RuCl_3_·*x*H_2_O, and (d) K_2_PtCl_4_ + Na_2_PdCl_4_.


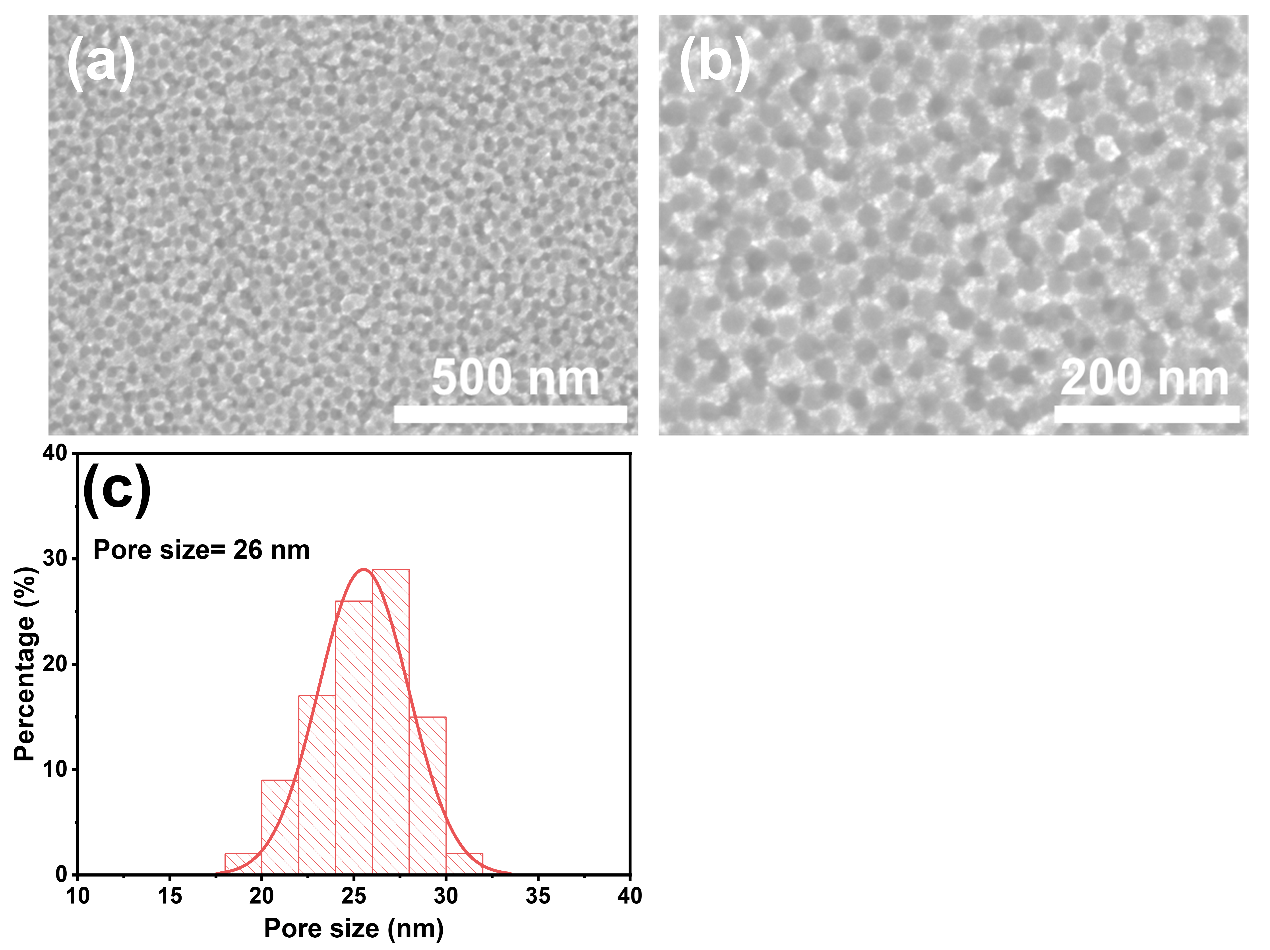


**Figure S2.** (a, b) Top-view SEM images of m-Pt film prepared using block copolymer PS_18000_-*b*-PEO_7500_. (c) Pore size distribution of m-Pt film prepared using block copolymer PS_18000_-*b*-PEO_7500_.


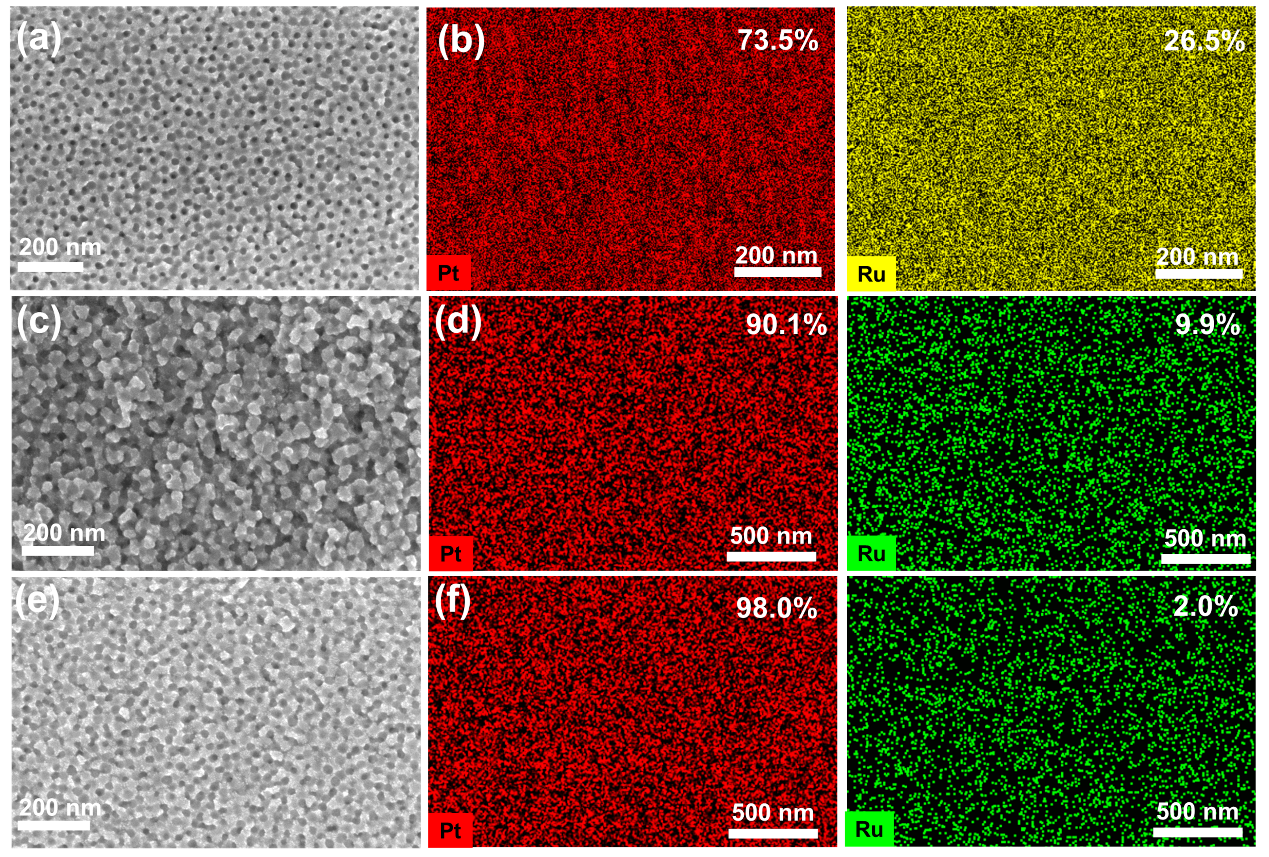


**Figure S3.** (a) Top-view SEM image and (b) EDS mapping images of m-Pt-Ru film with a feeding ratio of Pt:Ru = 1:1. (c) Top-view SEM image and (d) EDS mapping images of m-Pt-Ru film with a feeding ratio of Pt: Ru = 3:1. (e) Top-view SEM image and (f) EDS mapping images of m-Pt-Ru film with a feeding ratio of Pt: Ru = 9:1. All the films were prepared using block copolymer PS_18000_-*b*-PEO_7500_.


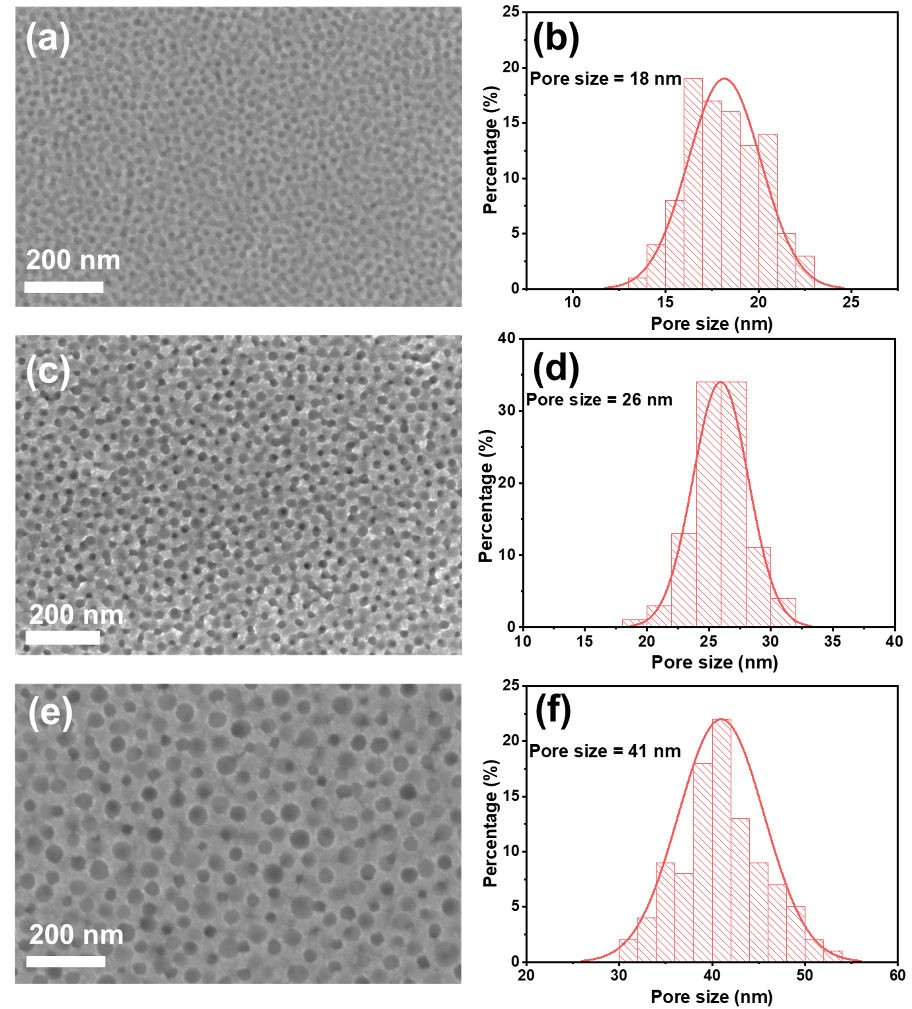


**Figure S4.** (a, b) SEM image (a) and pore size (b) of m-Pt-Ru film prepared using block polymer PS_10000_-*b*-PEO_4100_. (c, d) SEM image (c) and pore size (d) of m-Pt-Ru film prepared using block polymer PS_18000_-*b*-PEO_7500_. (e, f) SEM image (e) and pore size (f) of m-Pt-Ru film prepared using block polymer PS_63000_-*b*-PEO_26000_.


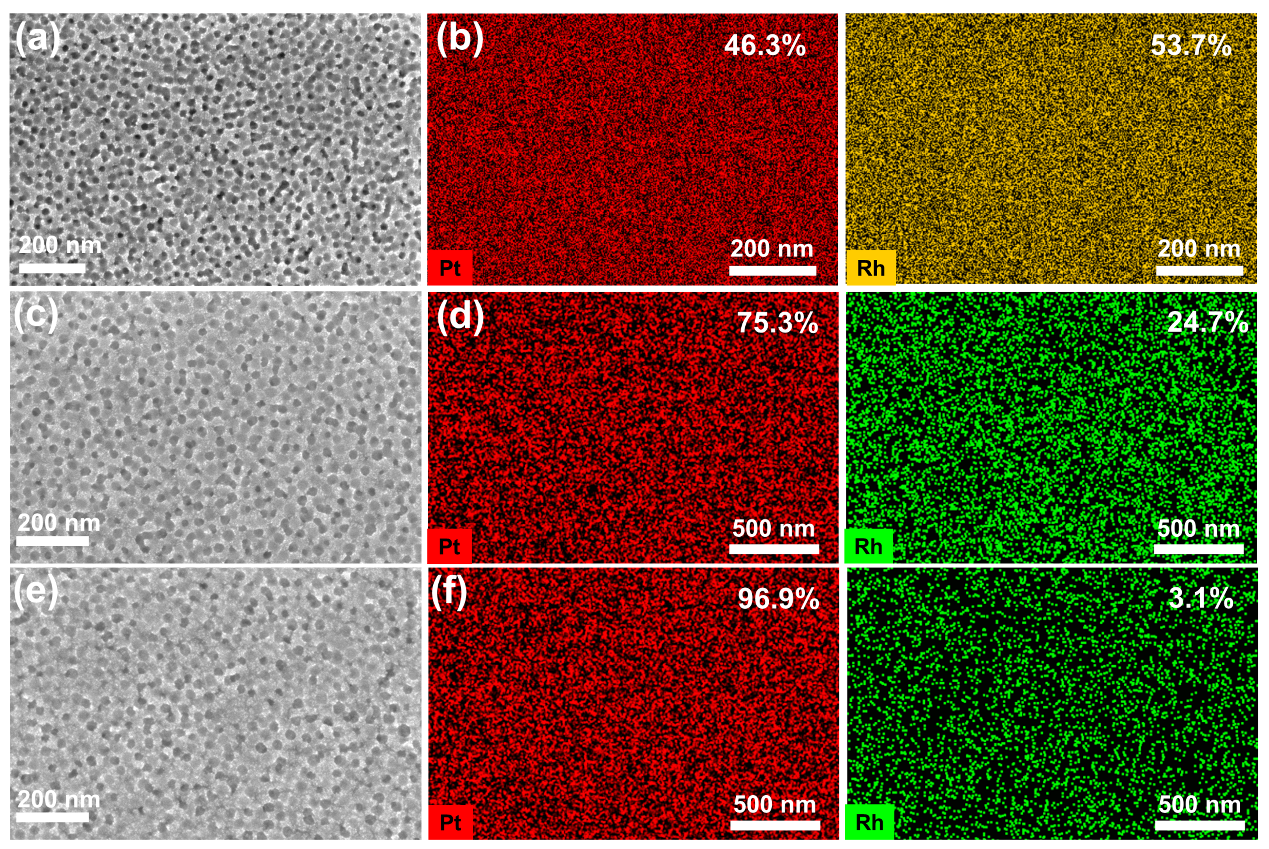


**Figure S5.** (a) Top-view SEM image and (b) EDS mapping images of m-Pt-Rh film with a feeding ratio of Pt:Rh = 1:1. (c) Top-view SEM image and (d) EDS mapping images of m-Pt-Rh film with a feeding ratio of Pt:Rh = 3:1. (e) Top-view SEM image and (f) EDS mapping images of m-Pt-Rh film with a feeding ratio of Pt:Rh = 9:1. All the films were prepared using block copolymer PS_18000_-*b*-PEO_7500_.


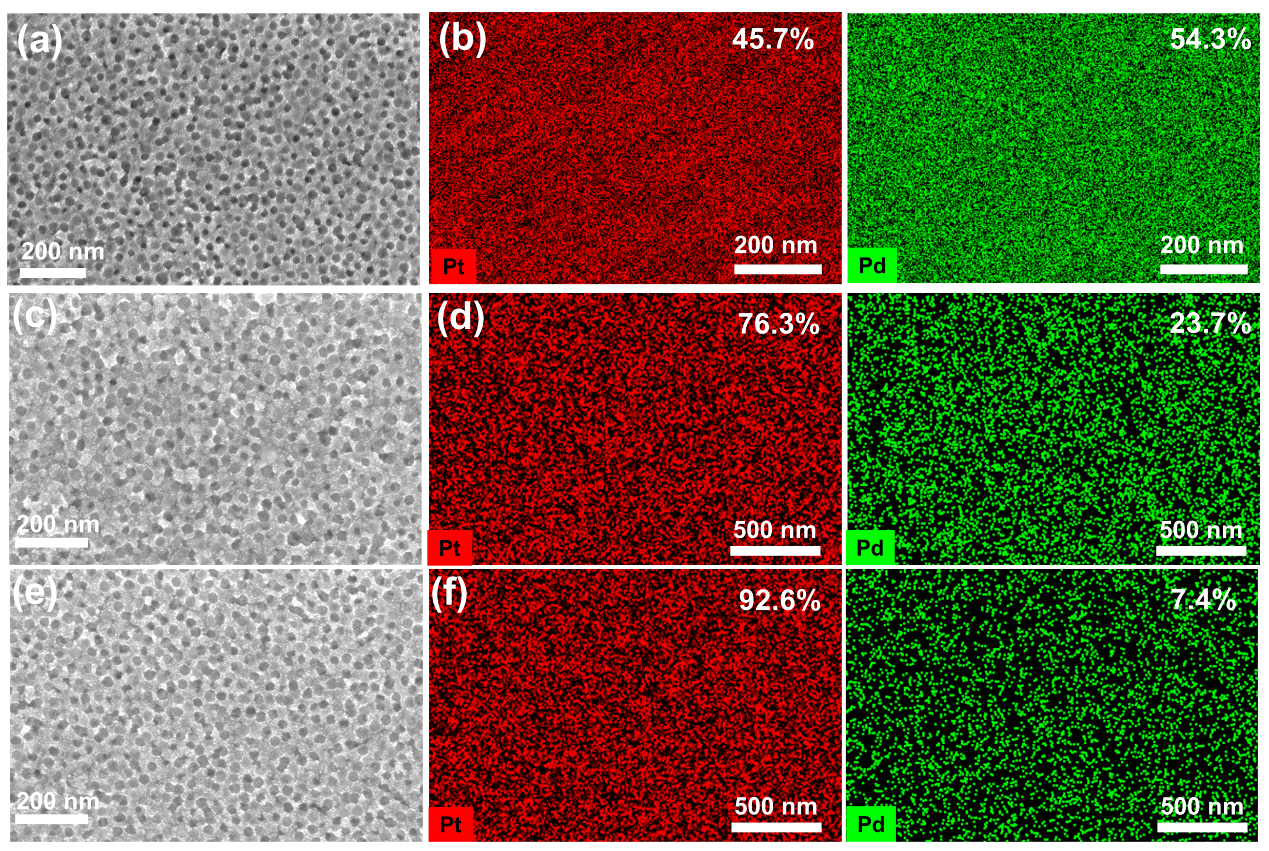


**Figure S6.** (a) Top-view SEM image and (b) EDS mapping images of m-Pt-Pd film with a feeding ratio of Pt:Pd = 1:1. (c) Top-view SEM image and (d) EDS mapping images of m-Pt-Pd film with a feeding ratio of Pt:Pd = 3:1. (e) Top-view SEM image and (f) EDS mapping images of m-Pt-Pd film with a feeding ratio of Pt: Pd = 9:1. All the films were prepared using block copolymer PS_18000_-*b*-PEO_7500_.


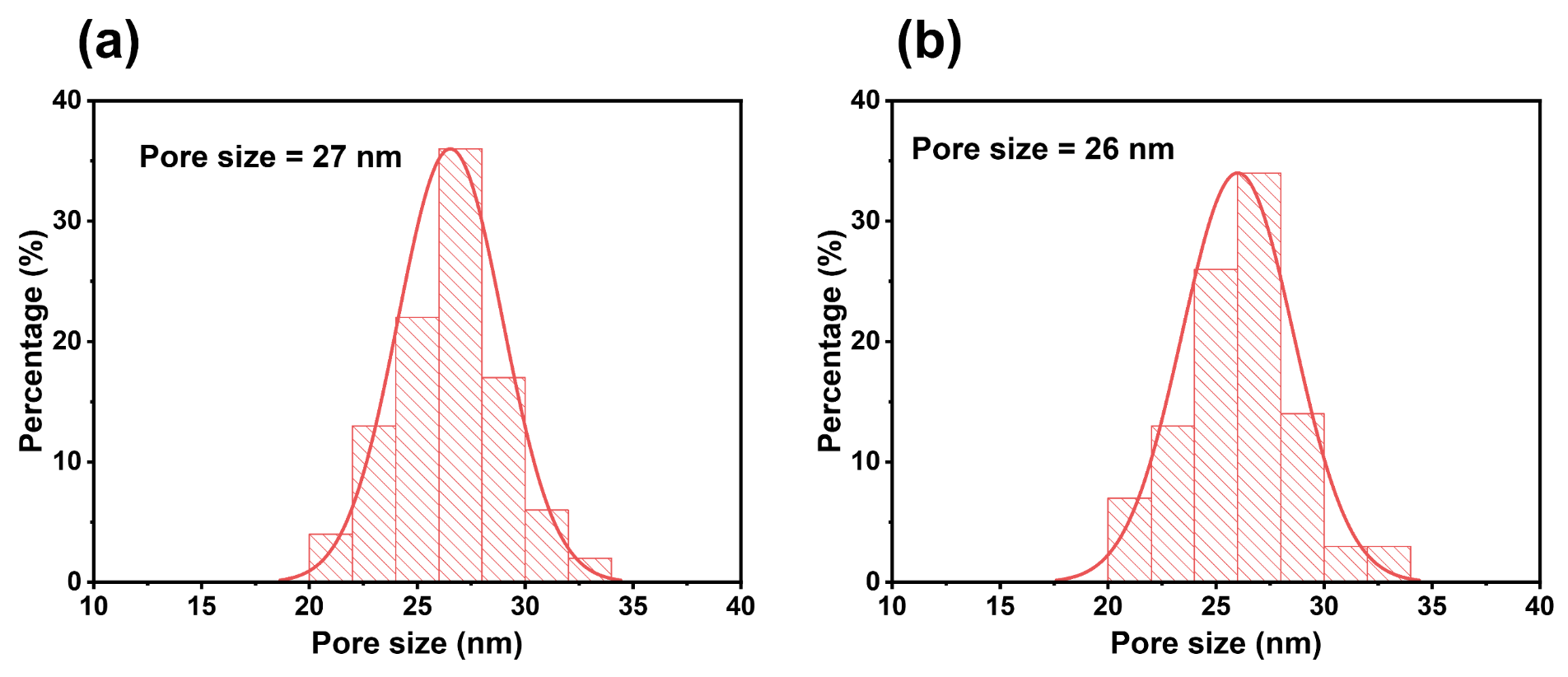


**Figure S7.** Pore sizes of (a) m-Pt-Pd and (b) m-Pt-Rh films prepared using block copolymer PS_18000_-*b*-PEO_7500_.


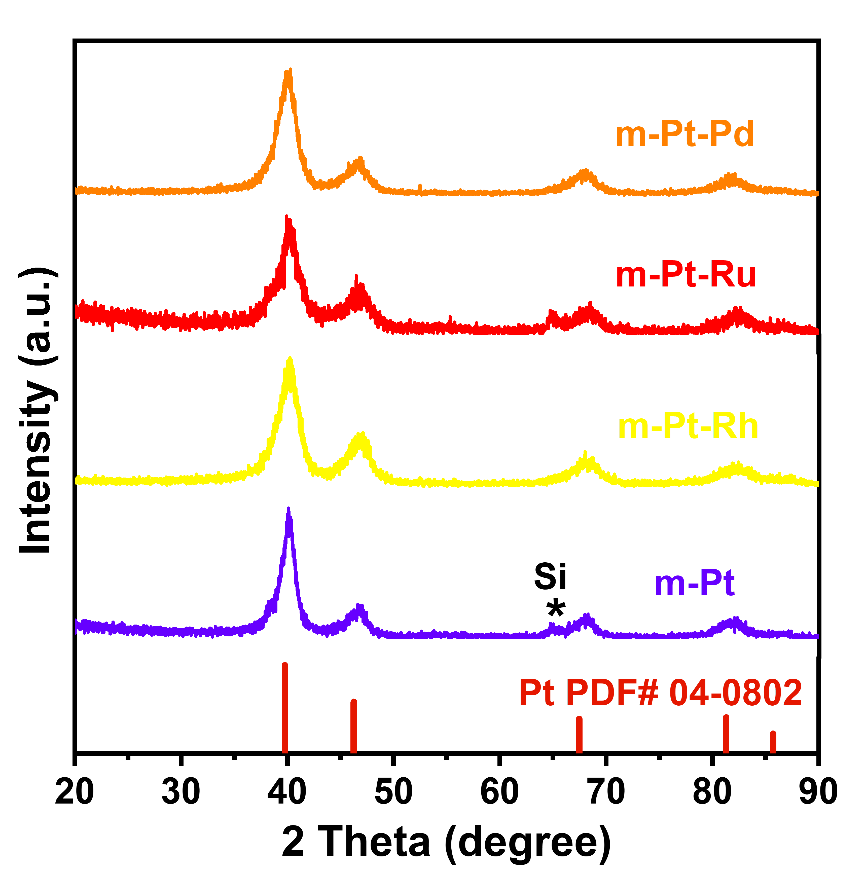


**Figure S8.** XRD patterns of m-Pt-M films with a similar atomic ratio of approximately Pt:M = 3:1, prepared using the block copolymer PS_18000_-*b*-PEO_7500_.


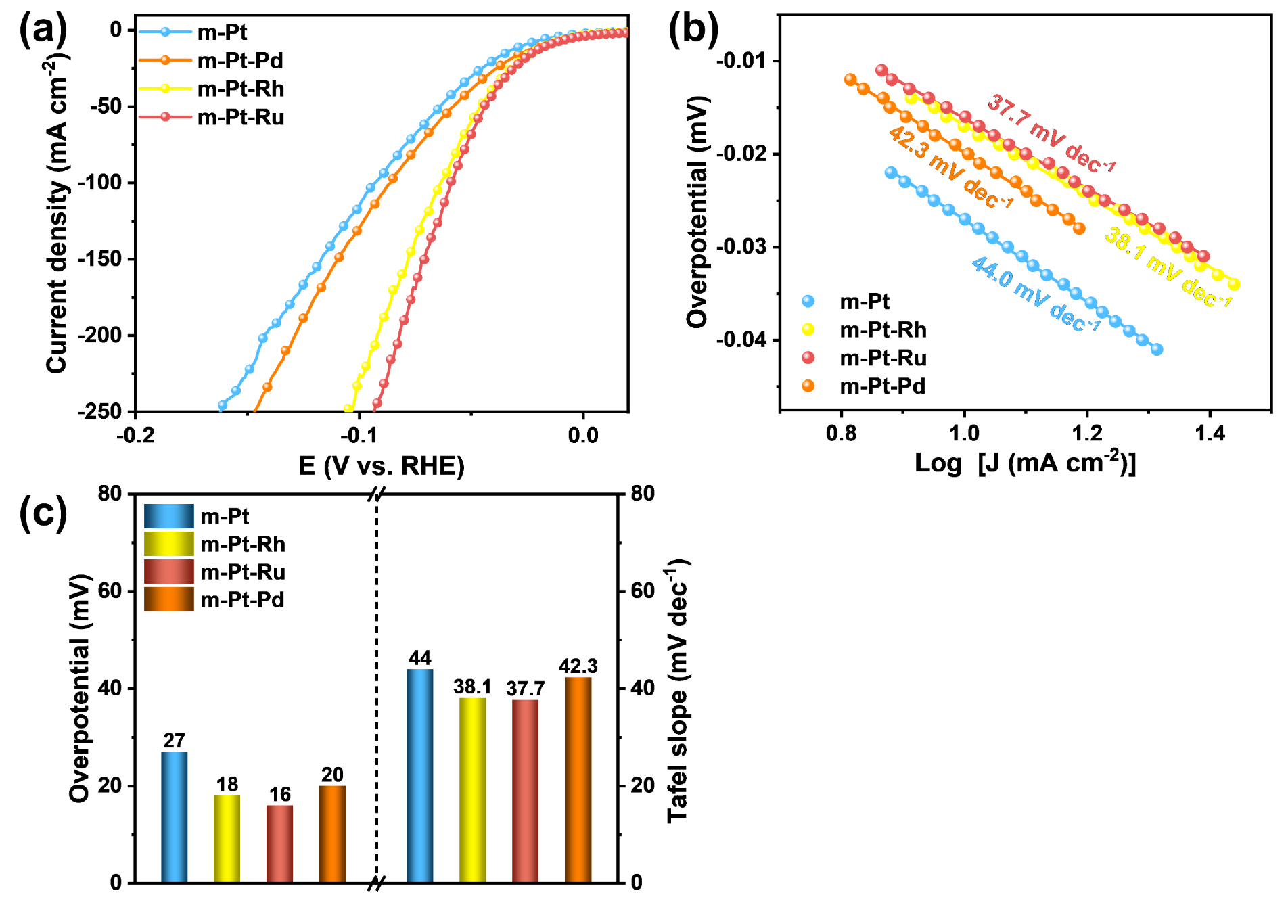


**Figure S9.** (a) LSV curves, (b) Tafel slopes, and (c) overpotentials and Tafel slopes comparison for m-Pt and m-Pt-Rh, m-Pt-Ru, and m-Pt-Pd with a similar atomic ratio of approximately Pt:M = 3:1, prepared using the block copolymer PS_18000_-*b*-PEO_7500_.


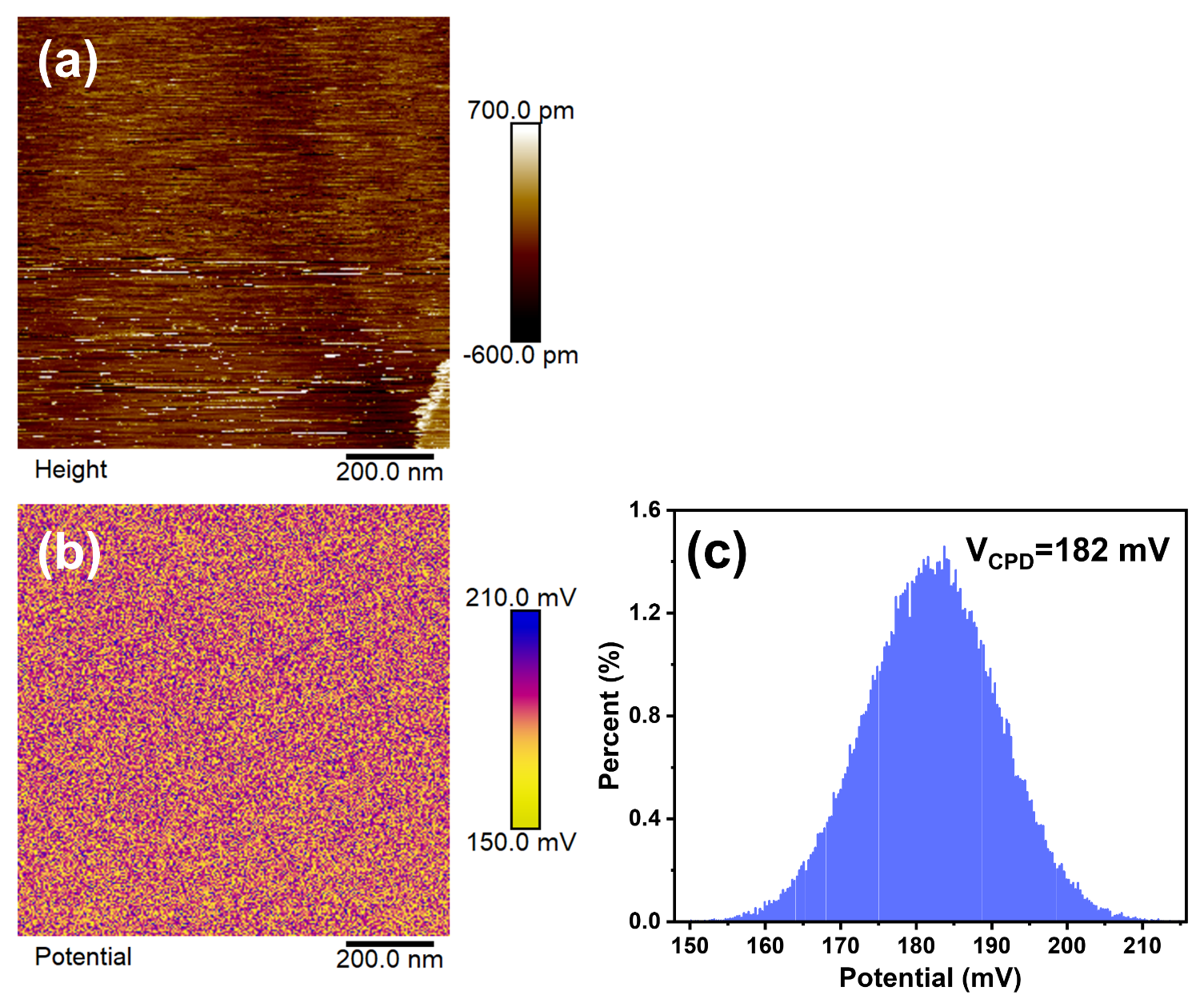


**Figure S10.** (a) KPFM height, (b) corresponding potential images, and (c) source data histogram in (b) of highly oriented pyrolytic graphite.


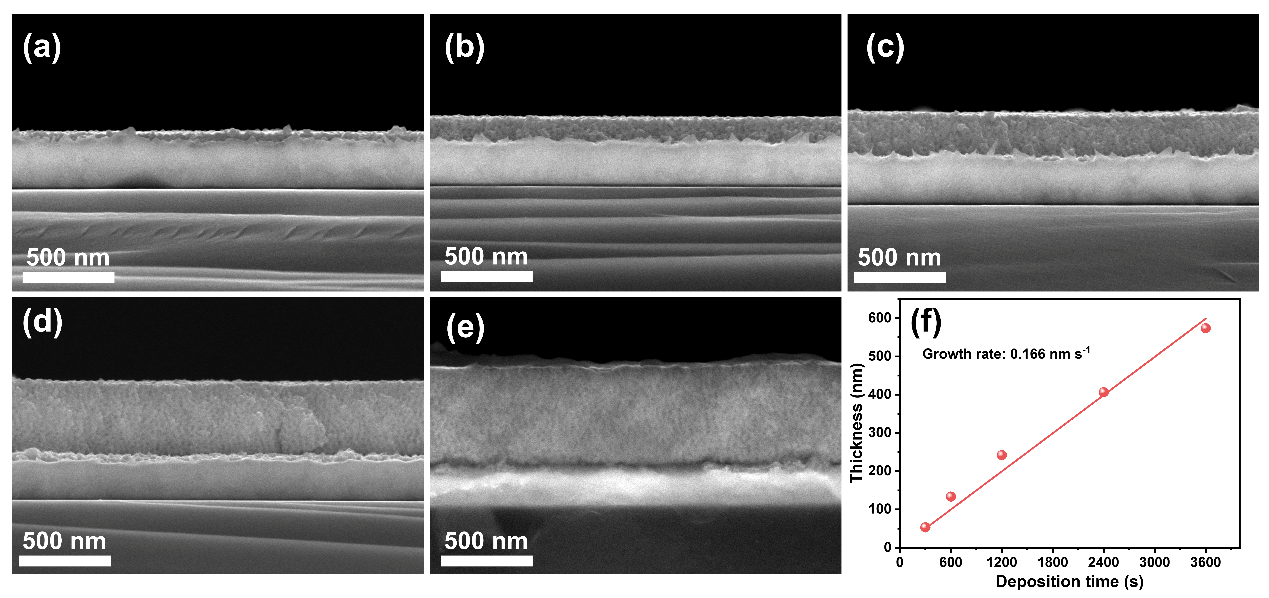


**Figure S11.** (a-e) Cross-sectional SEM images of m-Pt-Ru (with an atomic ratio of approximately Pt:Ru = 3:1) with different deposition times ((a) 300 s, (b) 600 s, (c) 1200 s, (d) 2400 s, and (e) 3600 s, respectively). All the films were prepared using the block copolymer PS_18000_-*b*-PEO_7500_. (f) Relationship between the m-Pt-Ru thickness and the deposition time.


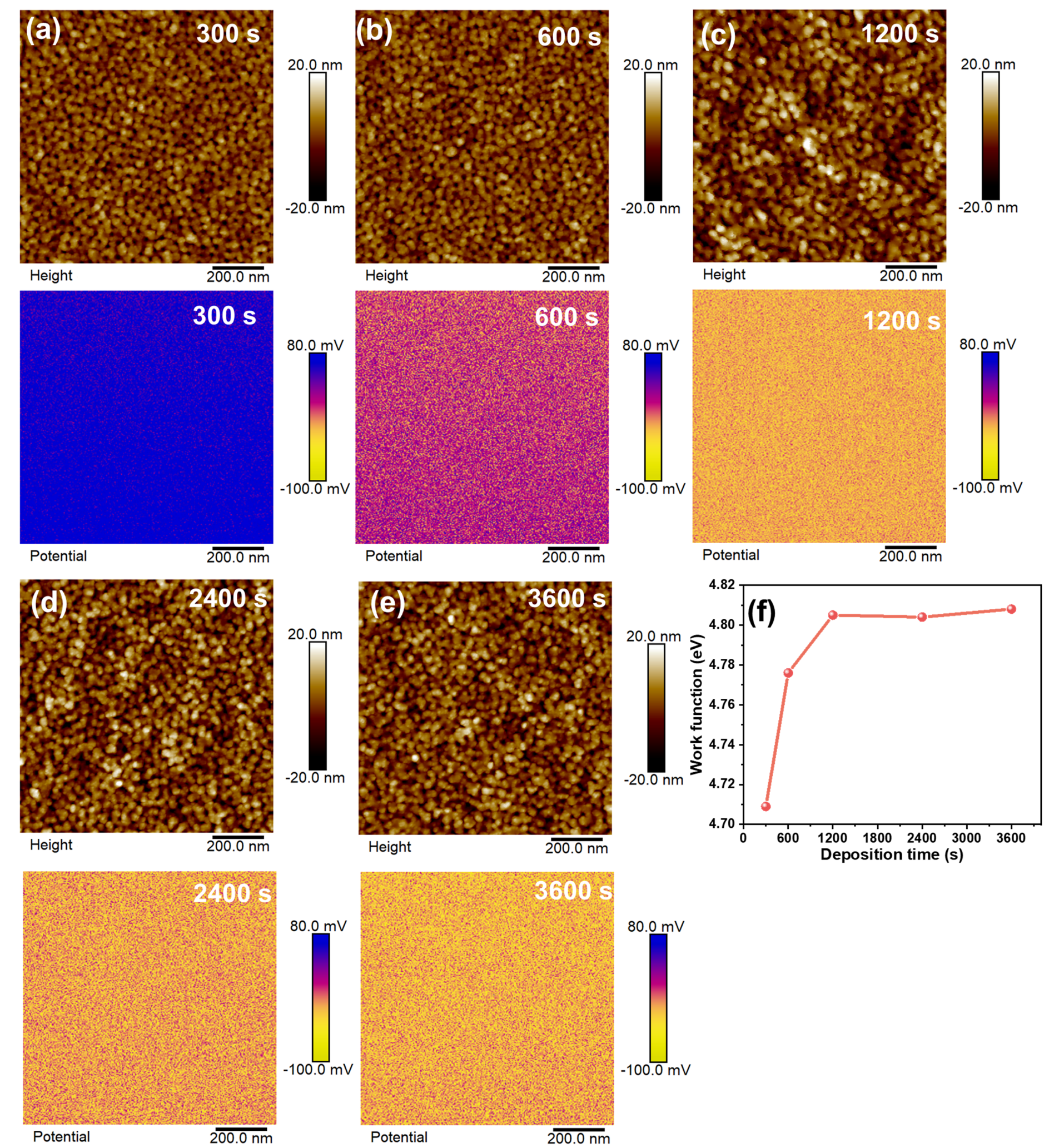


**Figure S12.** (a-e) Height images (top) and potential images (bottom) of m-Pt-Ru films (with an atomic ratio of approximately Pt:Ru = 3:1) prepared with varying deposition times. (f) Work functions of m-Pt-Ru films (with an atomic ratio of approximately Pt:Ru = 3:1) prepared with varying deposition times. All the films were prepared using the block copolymer PS_18000_-*b*-PEO_7500_.


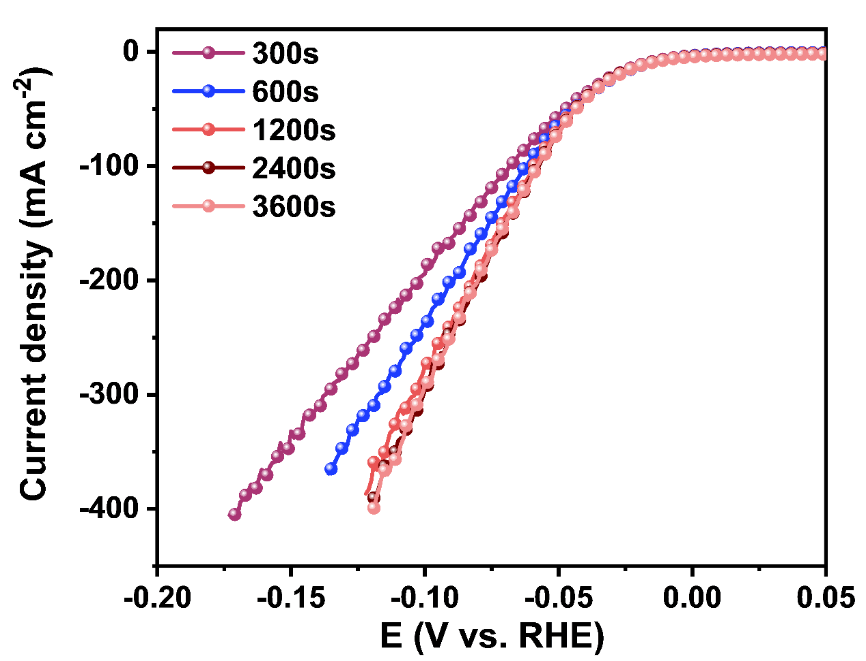


**Figure S13.** LSV curves of m-Pt-Ru films (with an atomic ratio of approximately Pt:Ru = 3:1) prepared with varying deposition times. All the films were prepared using the block copolymer PS_18000_-*b*-PEO_7500_.


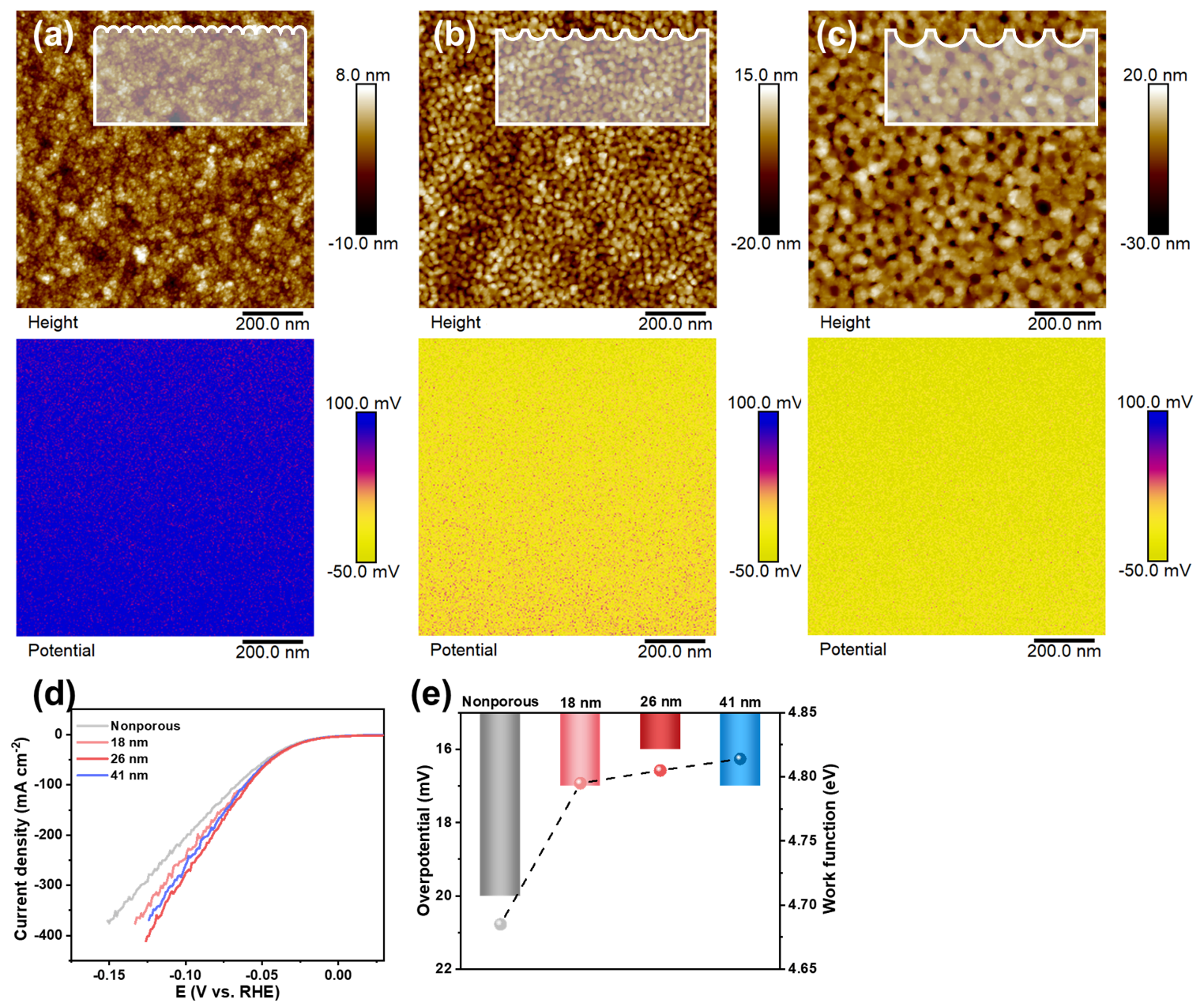


**Figure S14.** (a) Height images (upper) and potential images (down) of nonporous Pt-Ru film. (b) Height images (upper) and potential images (down) of m-Pt-Ru film with a pore size of 18 nm. (c) Height images (upper) and potential images (down) of m-Pt-Ru film with a pore size of 41 nm. Inset is the curvature schematic. (d) LSV curves and (e) overpotentials and work functions of nonporous Pt-Ru film and m-Pt-Ru film with different pore sizes. All the films have a similar atomic ratio of approximately Pt:Ru = 3:1.


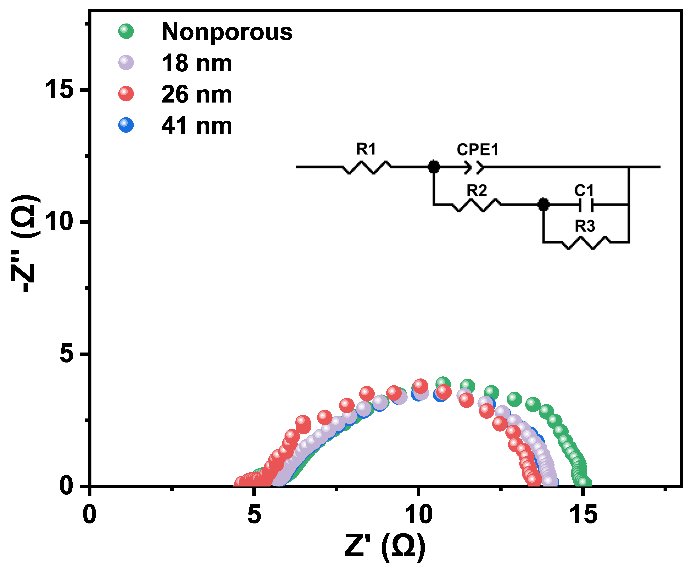


**Figure S15.** EIS spectra of nonporous Pt-Ru and m-Pt-Ru films with different pore sizes (18 nm, 26 nm, and 41 nm) at an overpotential of 30 mV. Insert is an equivalent fitted circuit.


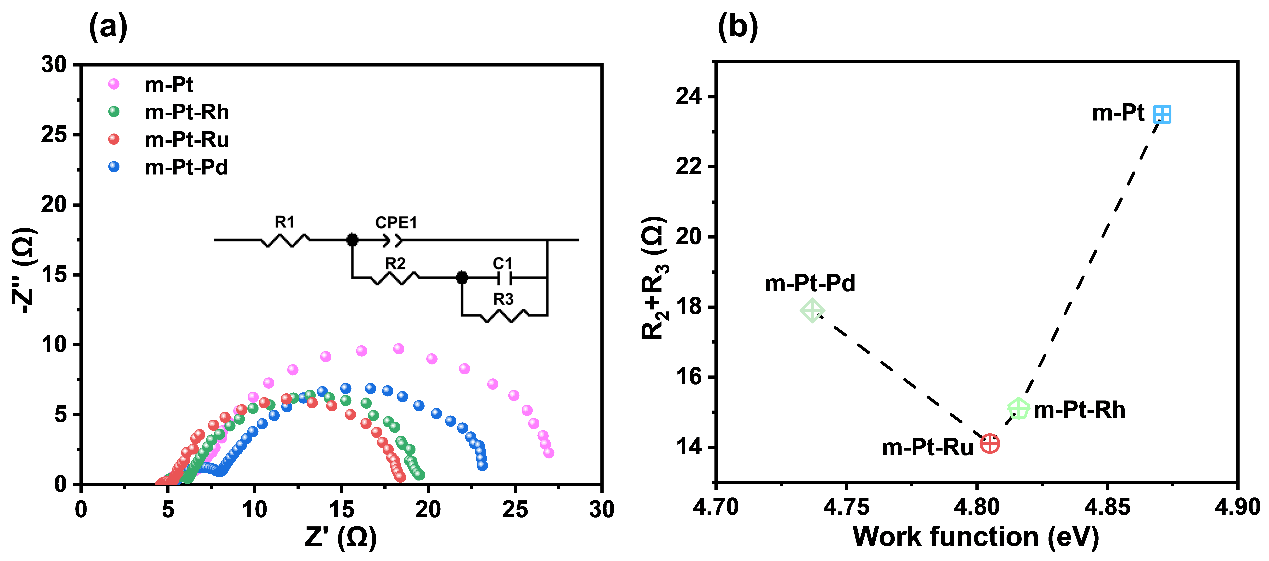


**Figure S16**. (a) EIS spectra of m-Pt and m-Pt-M (with a similar atomic ratio of approximately Pt:M = 3:1) at an overpotential of 20 mV. (b) Fitting results of EIS plotted against their work functions.


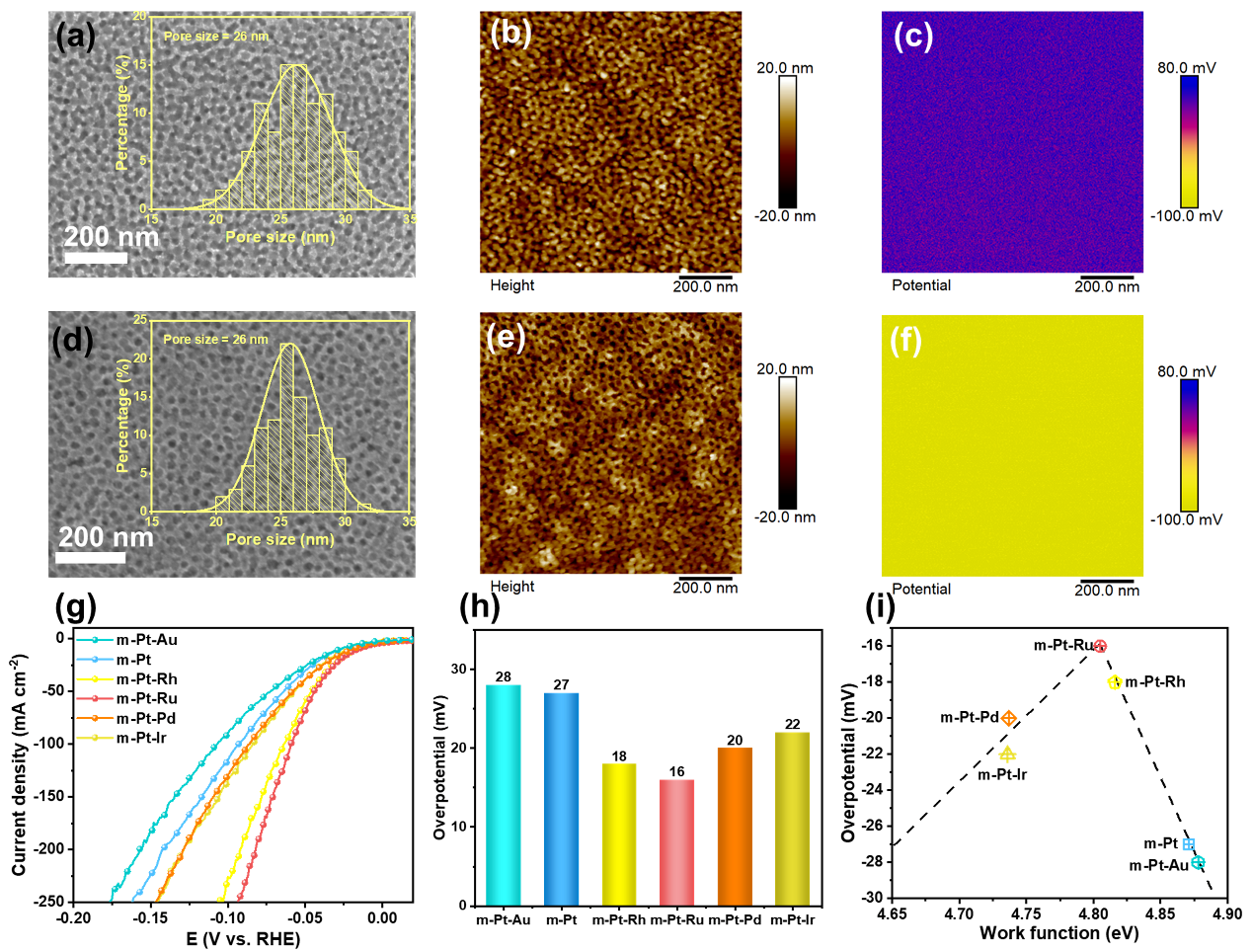


**Figure S17.** (a-c) SEM image (a), height image (b), and potential image (c) of m-Pt-Ir film. (d-ff) SEM image (d), height image (e), and potential image (f) of m-Pt-Au film. (d) LSV curves, (b) overpotentials, and (c) correlation of overpotentials with WFs for different samples.

Comment for **Figure S17:** As shown in **Figure S17**, the m-Pt-Ir and m-Pt-Au films exhibit identical pore sizes (26 nm) as the m-Pt and m-Pt-M films. Note that, despite Ir’s higher theoretical reduction potential compared to Pd/Rh/Ru, its metallic form exhibits pronounced electrodeposition constraints (*i.e.*, m-Pt-Ir (Pt_89_Ir_11_) film is prepared with a feeding ratio of Pt:Ir = 1:1). On the other hand, Au can be preferentially deposited, resulting that m-Pt-Au (Pt_68_Au_32_) is prepared with a feeding ratio of Pt:Au = 3:1. Furthermore, the HER performance and work functions of m-Pt-Ir and m-Pt-Au films follow the volcano plot trend observed in **Figure 1g**.


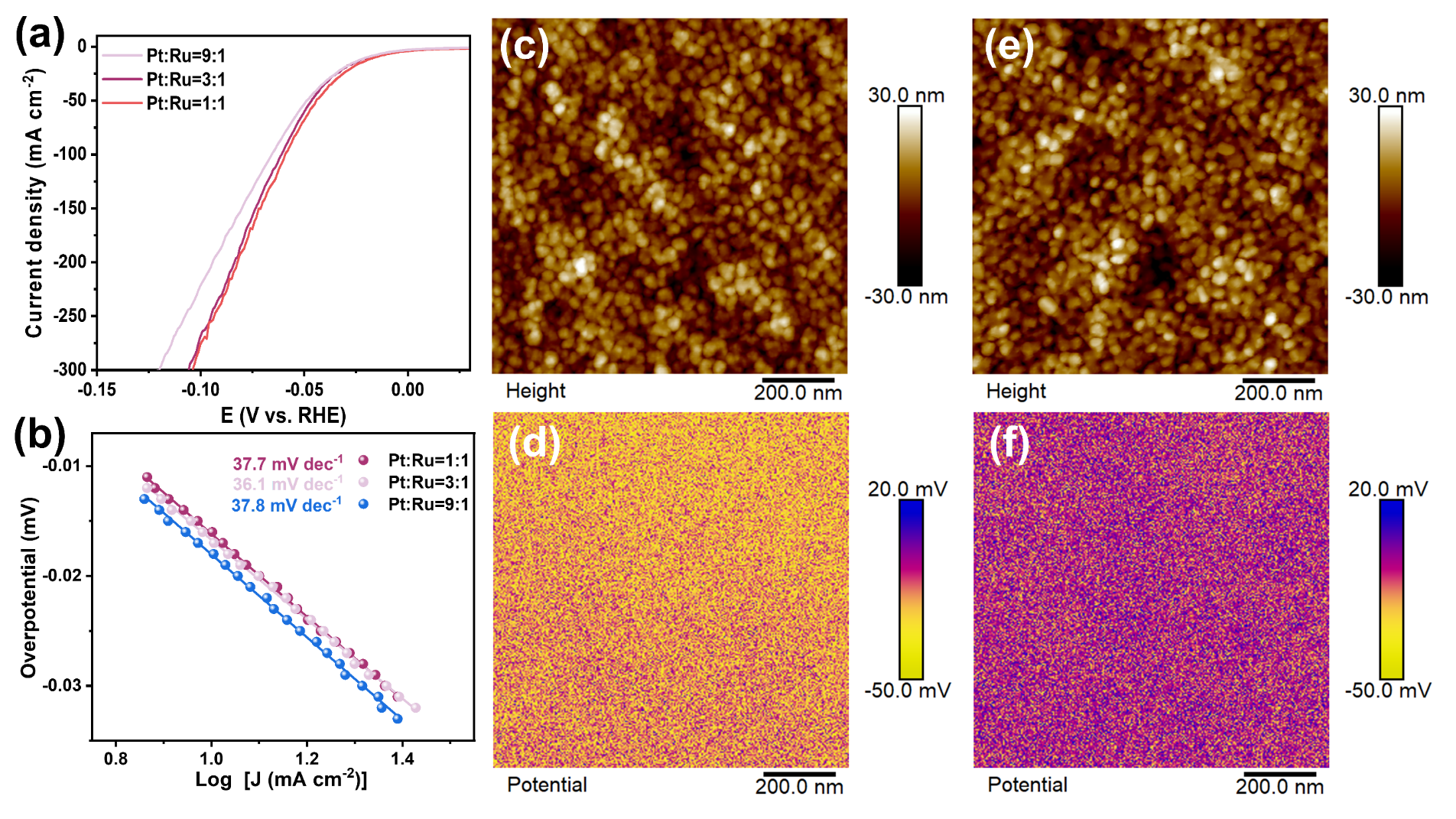


**Figure S18.** (a) LSV curves of m-Pt-Ru films with different feeding ratios. (b) Tafel slopes of m-Pt-Ru films with different feeding ratios (Pt:Ru = 9:1, 3:1, and 1:1). (c) Height image and (d) potential image of m-Pt-Ru film with a feeding ratio of Pt:Ru = 3:1. (e) Height image and (f) potential image of m-Pt-Ru film with a feeding ratio of Pt:Ru = 9:1.


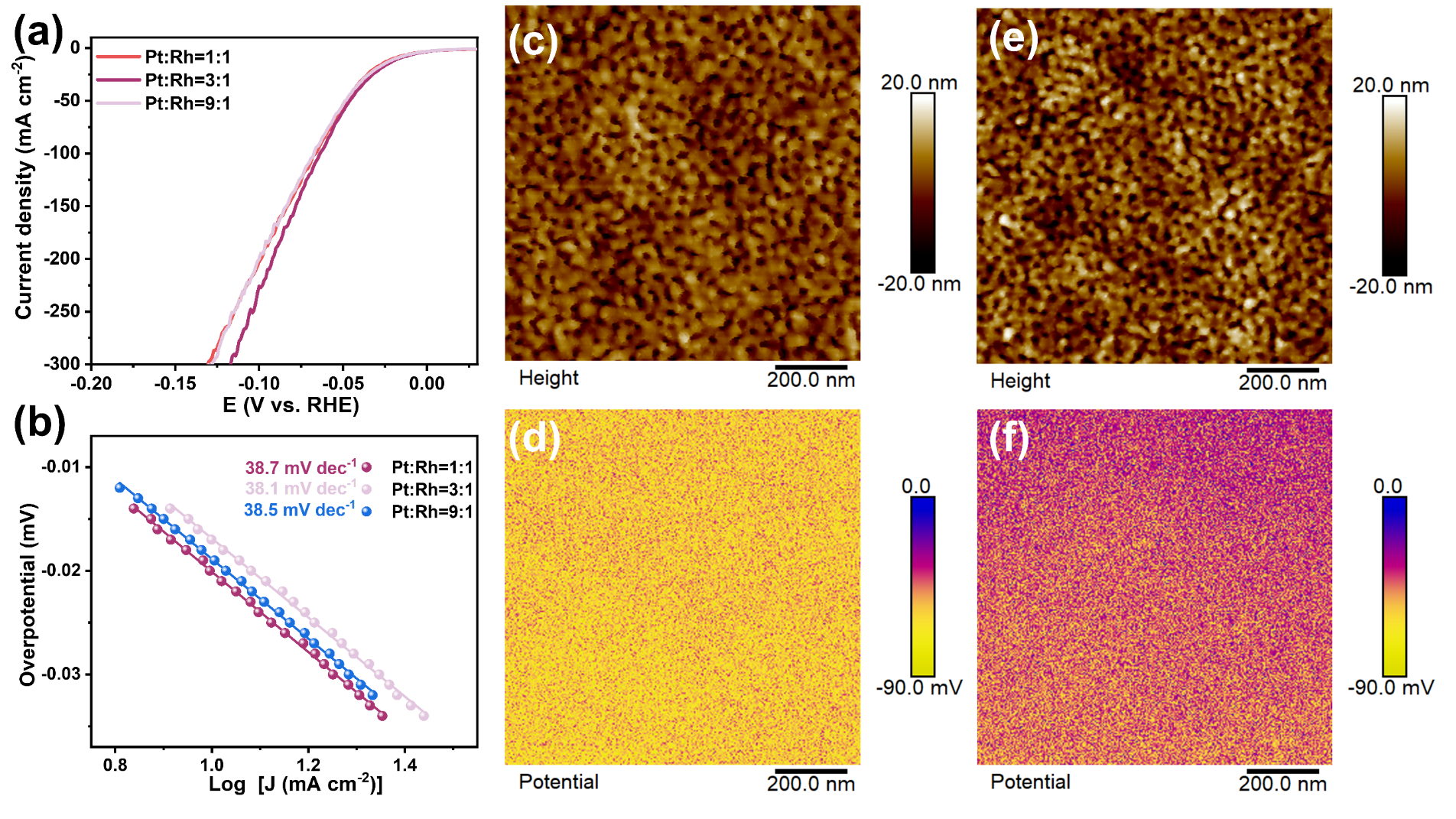


**Figure S19.** (a) LSV curves of m-Pt-Rh films with different feeding ratios. (b) Tafel slopes of m-Pt-Rh films with different feeding ratios (Pt:Rh = 9:1, 3:1, and 1:1). (c) Height image and (d) potential image of m-Pt-Rh film with a feeding ratio of Pt:Rh = 1:1. (e) Height image and (f) potential image of m-Pt-Rh film with a feeding ratio of Pt: Rh = 9:1.


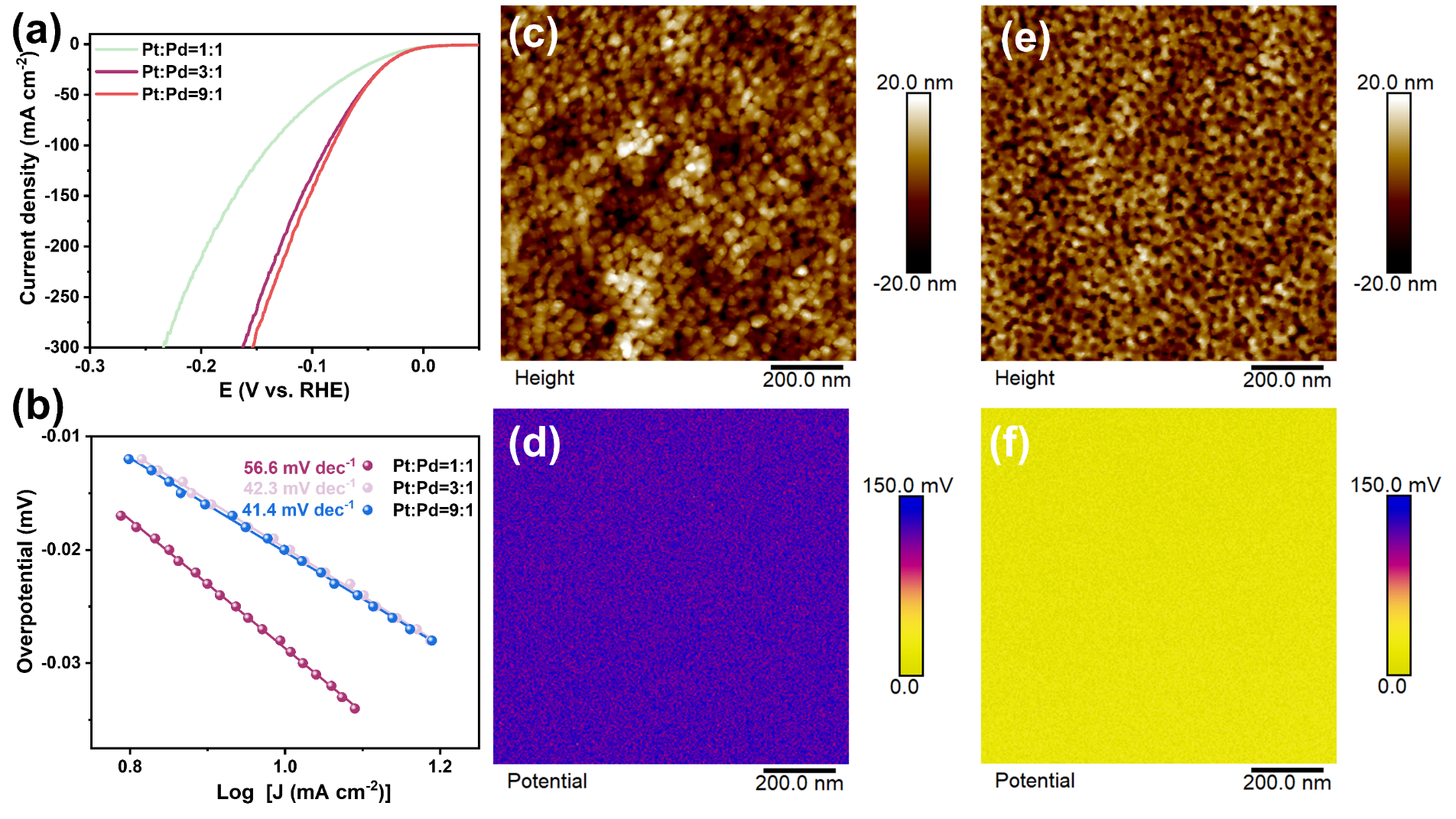


**Figure S20.** (a) LSV curves of m-Pt-Pd films with different feeding ratios (Pt:Pd = 9:1, 3:1, and 1:1). (b) Tafel slopes of m-Pt-Pd films with different feeding ratios (Pt:Pd = 9:1, 3:1, and 1:1). (c) Height image and (d) potential image of m-Pt-Pd film with a feeding ratio of Pt:Pd = 1:1. (e) Height image and (f) potential image of m-Pt-Pd film with a feeding ratio of Pt: Pd = 9:1.


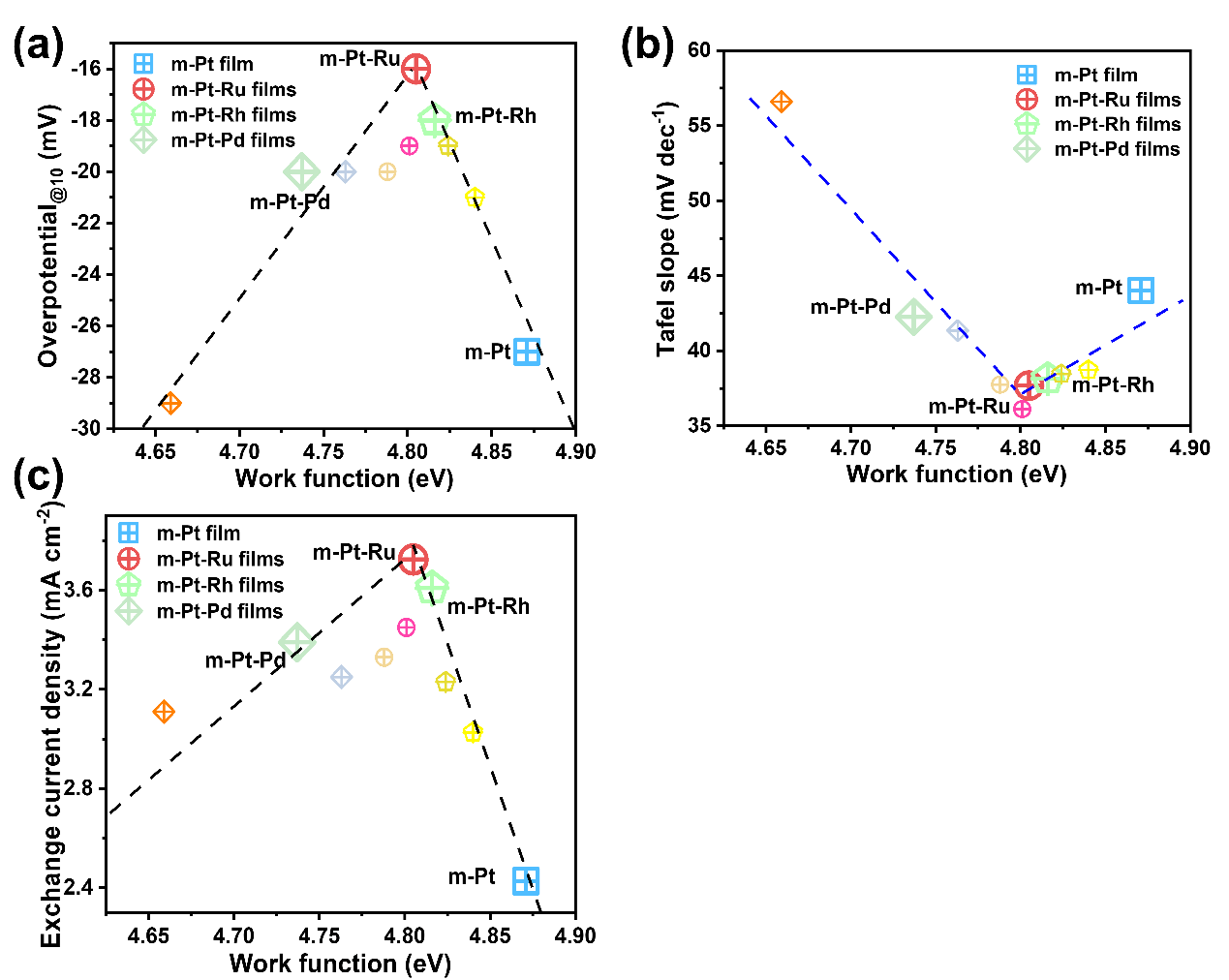


**Figure S21.** (a) Correlation of HER overpotentials, (b) Tafel slopes, and (c) exchange current density of m-Pt-M films with their work functions. Large-sized markers represent typical m-Pt and m-Pt-M films with a similar atomic ratio of approximately Pt:M = 3:1.


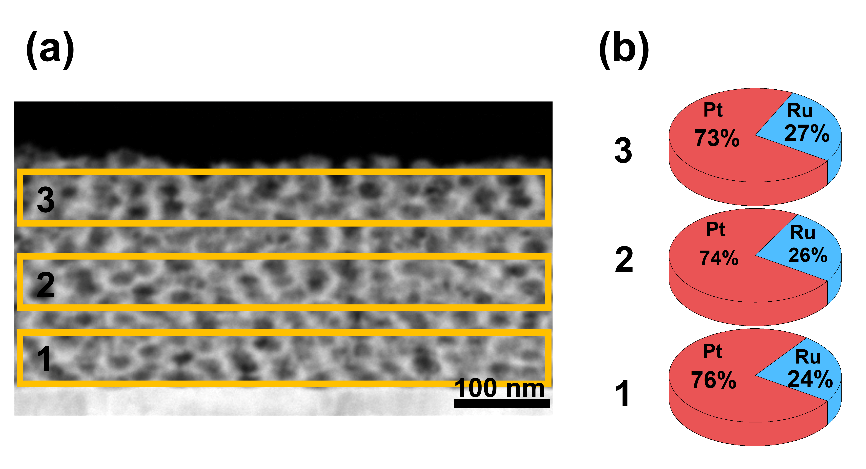


**Figure S22.** (a) HAADF image of m-Pt-Ru and (b) corresponding atomic ratio at selected areas (*i.e.*, 1, 2, and 3).


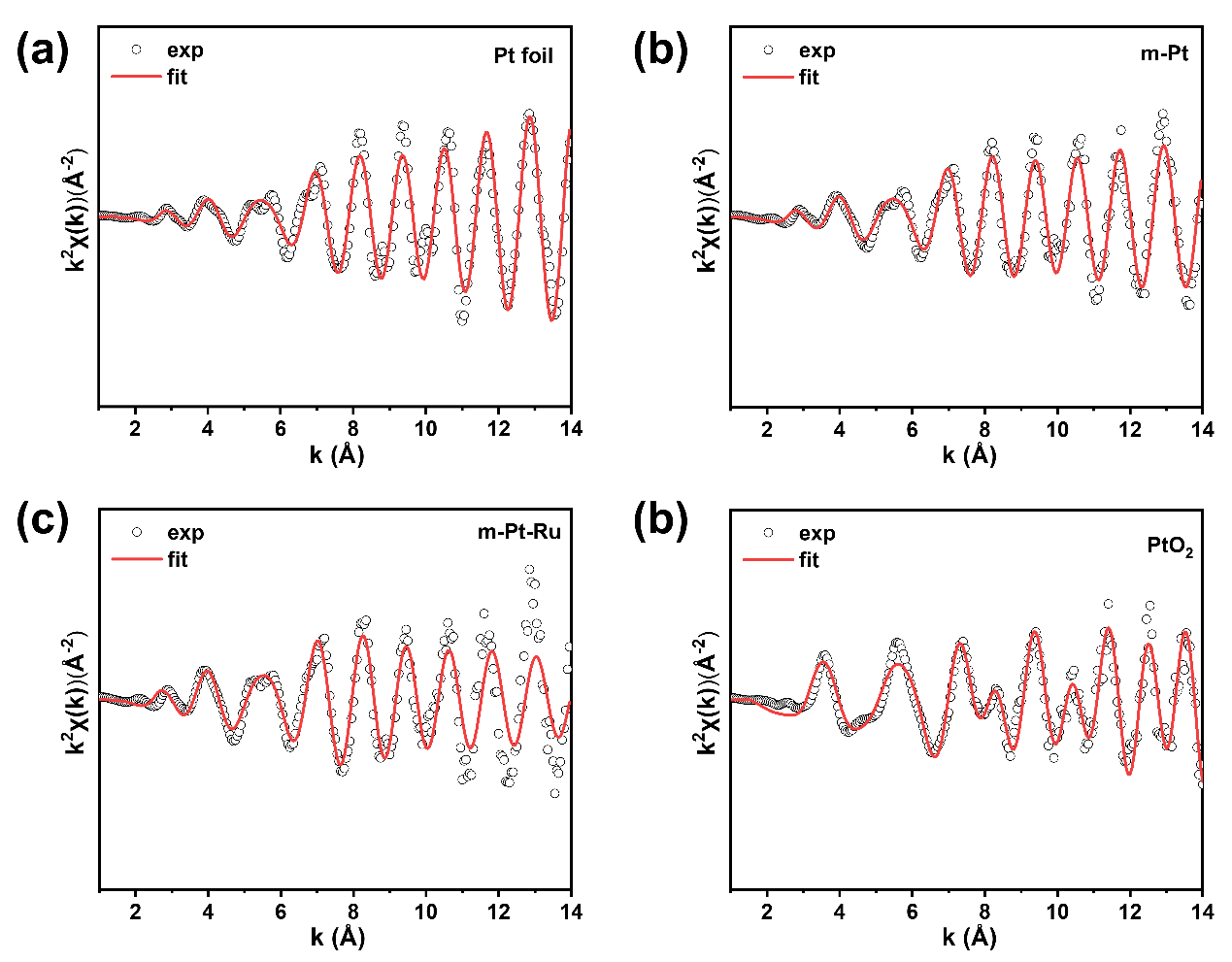


**Figure S23.** *k*^2^-weighted k-space Pt L_3_-edge experimental and fitting spectra of (a) Pt foil, (b) m-Pt, (c) m-Pt-Ru, and (d) PtO_2_.


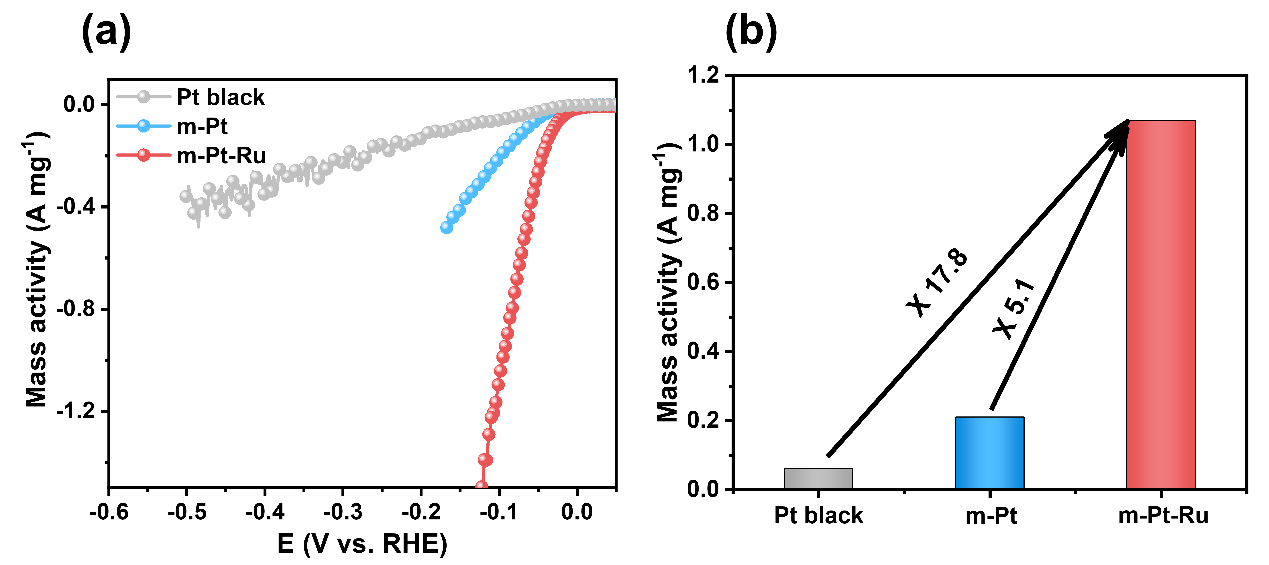


**Figure S24.** (a) Mass activity curves of m-Pt, m-Pt-Ru, and Pt black. (d) Comparison of mass activity at an overpotential of 100 mV.


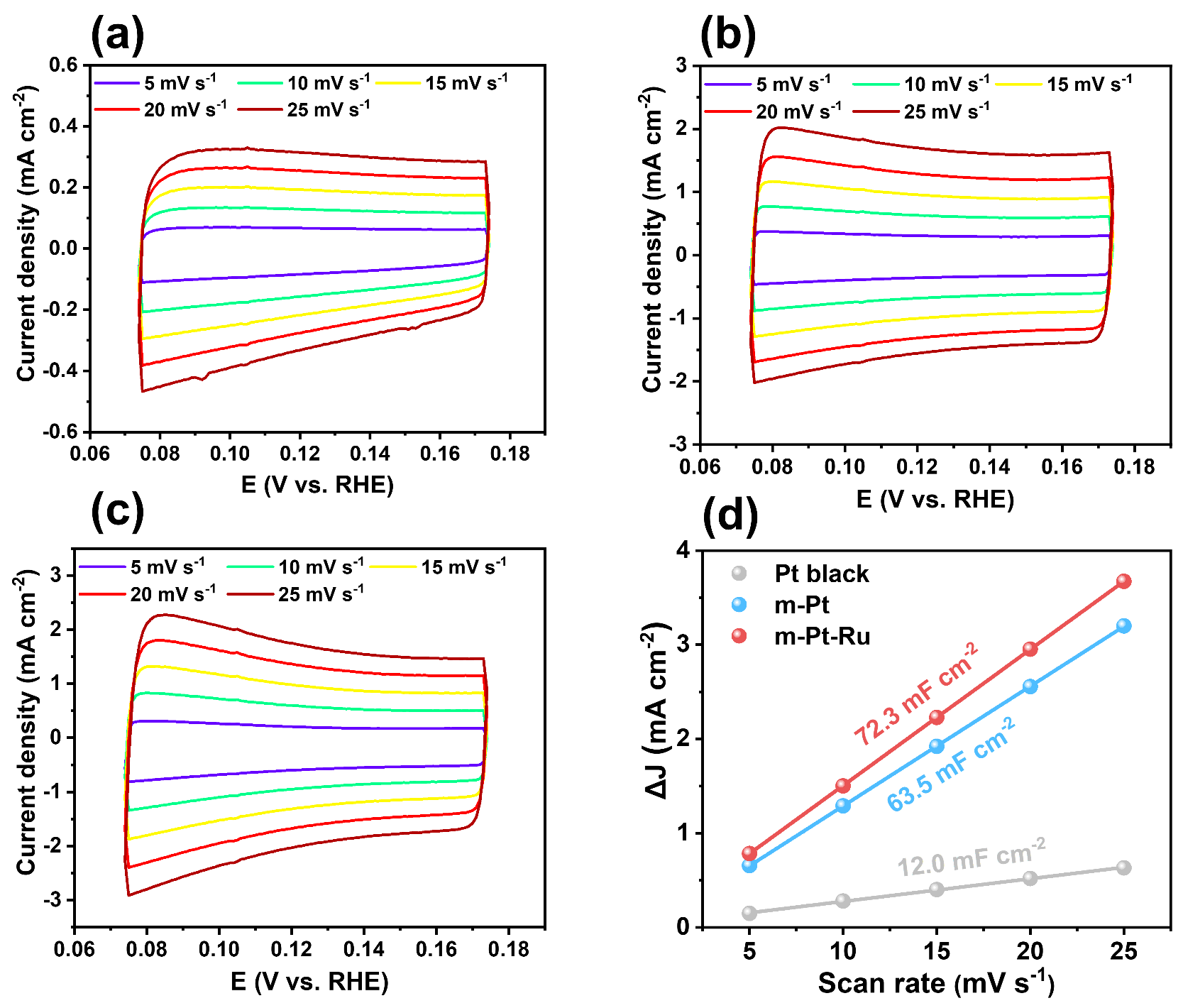


**Figure S25.** (a-c) CV curves of (a) Pt black, (b) m-Pt, and (d) m-Pt-Ru at different scan rates. (d) C_dl_ values of different samples.


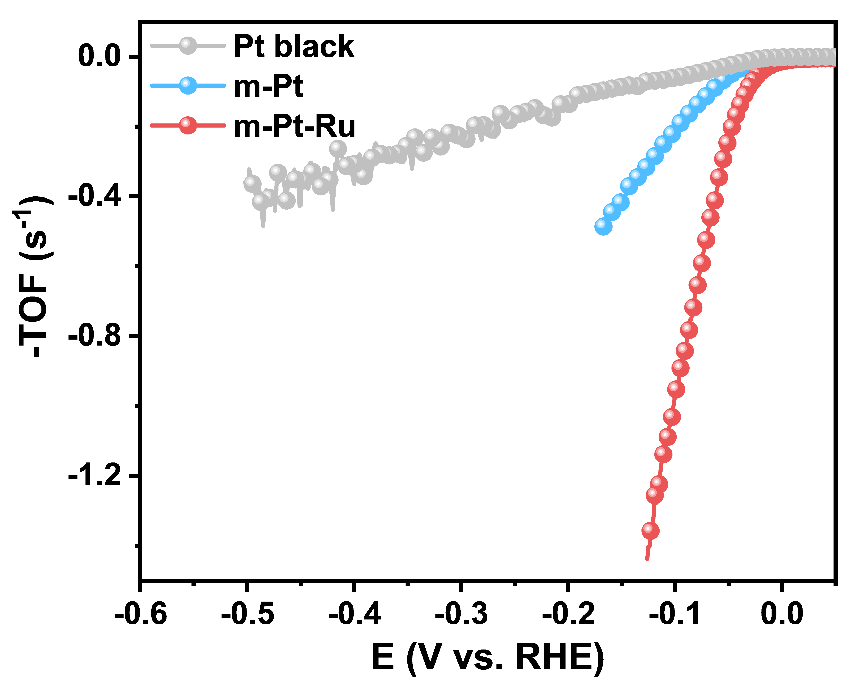


**Figure S26.** TOF curves of m-Pt-Ru, m-Pt, and Pt black.


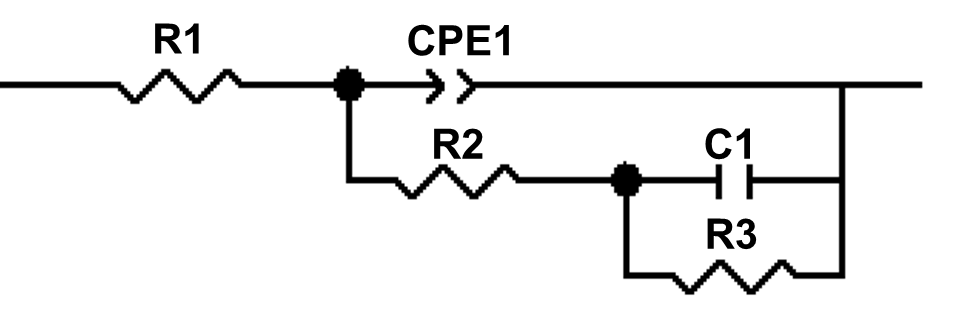


**Figure S27.** An equivalent fitted circuit for EIS.


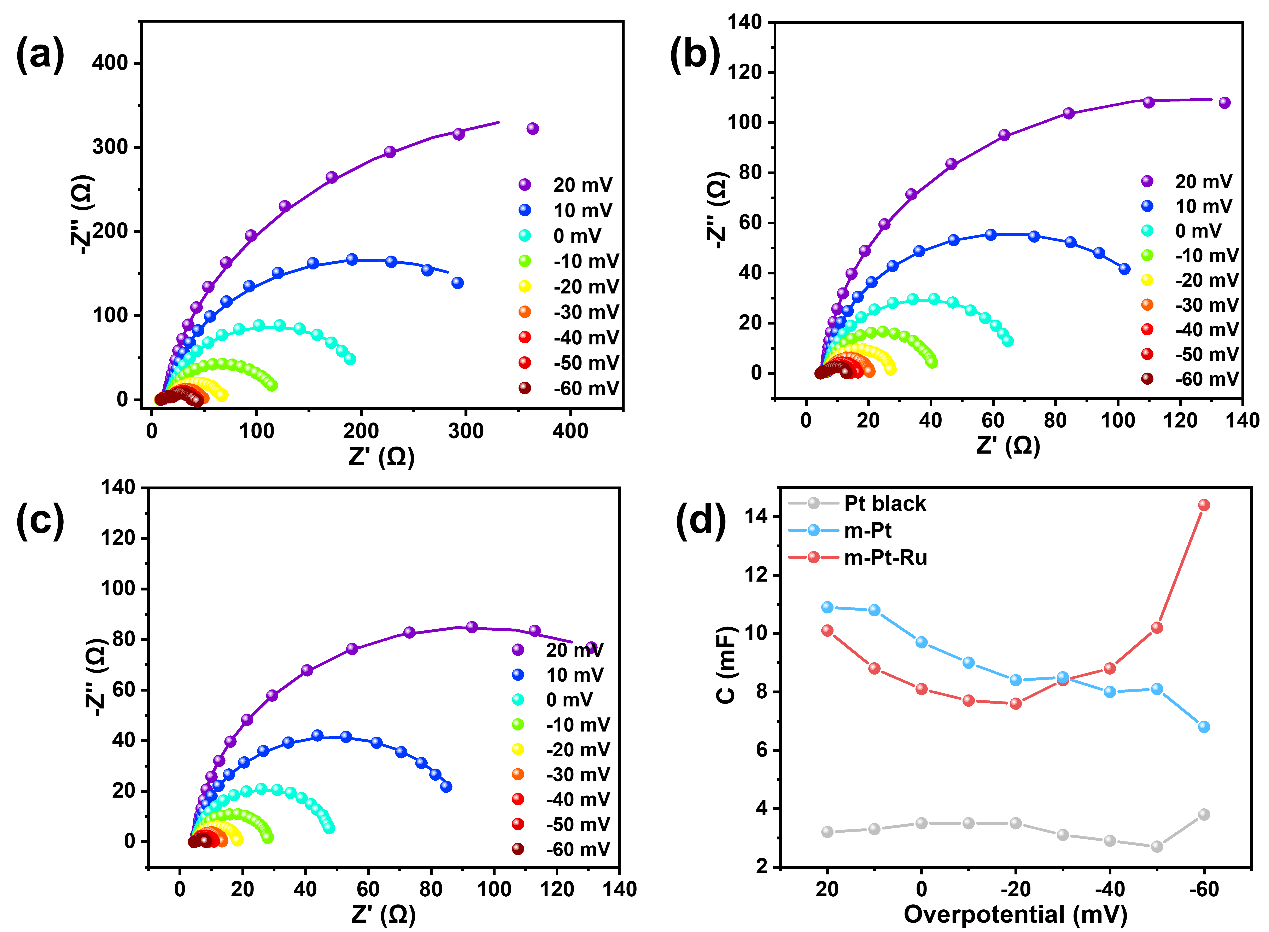


**Figure S28.** EIS spectra of (a) Pt black, (b) m-Pt, and (c) m-Pt-Ru. (d) Fitted Cφ values for Pt black, m-Pt, and m-Pt-Ru.


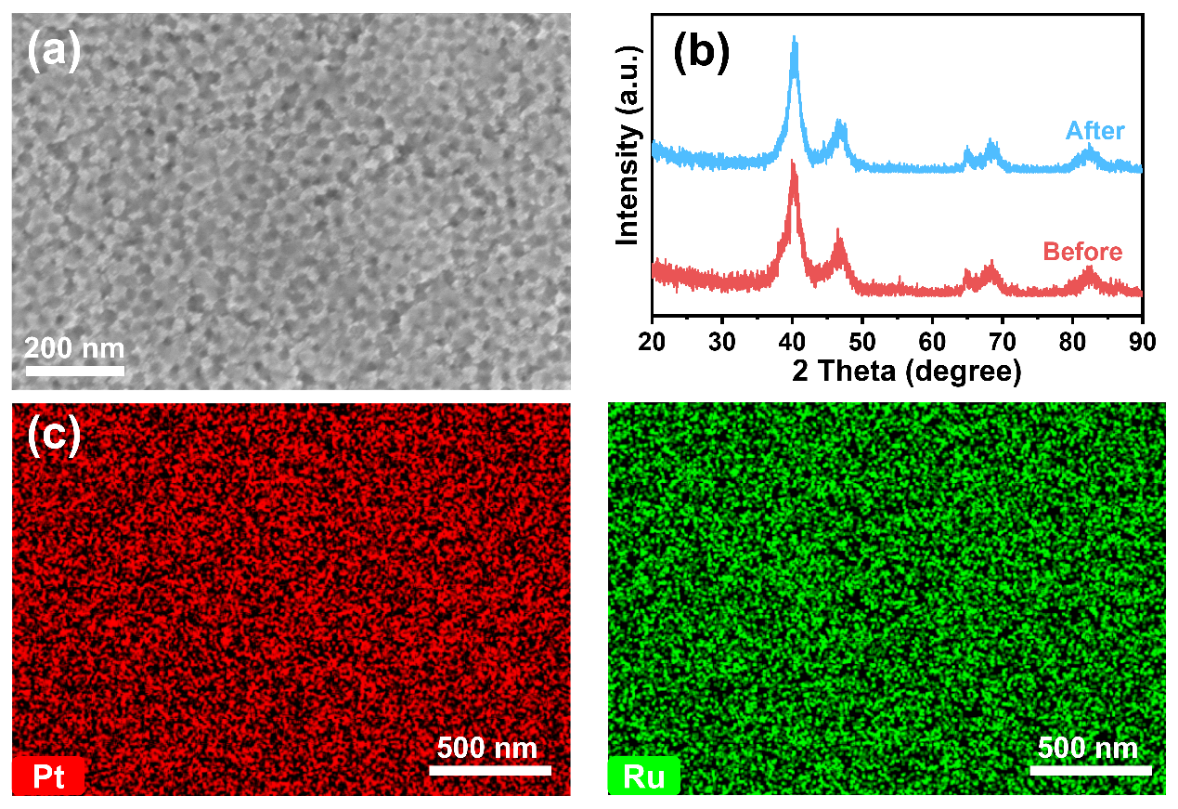


**Figure S29.** (a) SEM image of m-Pt-Ru after stability test. (b) XRD patterns m-Pt-Ru before and after the stability test. (c) SEM-EDS mapping images of m-Pt-Ru after stability test.


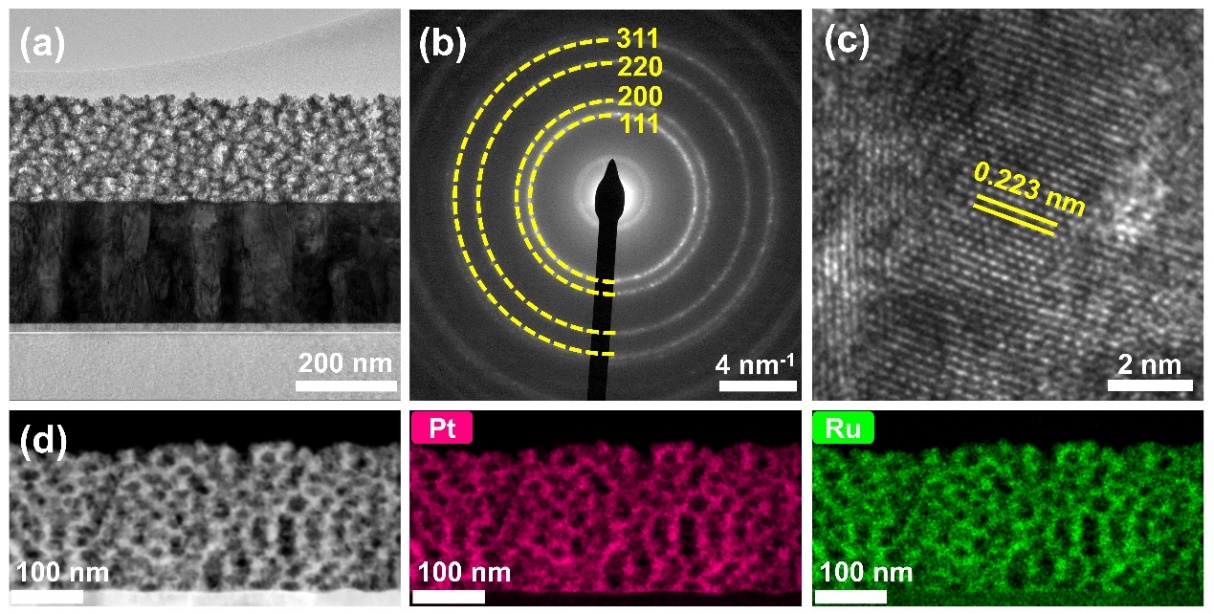


**Figure S30.** (a) TEM image, (b) SAED pattern, (c) HRTEM image, and (g) HAADF image and corresponding EDS mapping images of m-Pt-Ru after stability test.


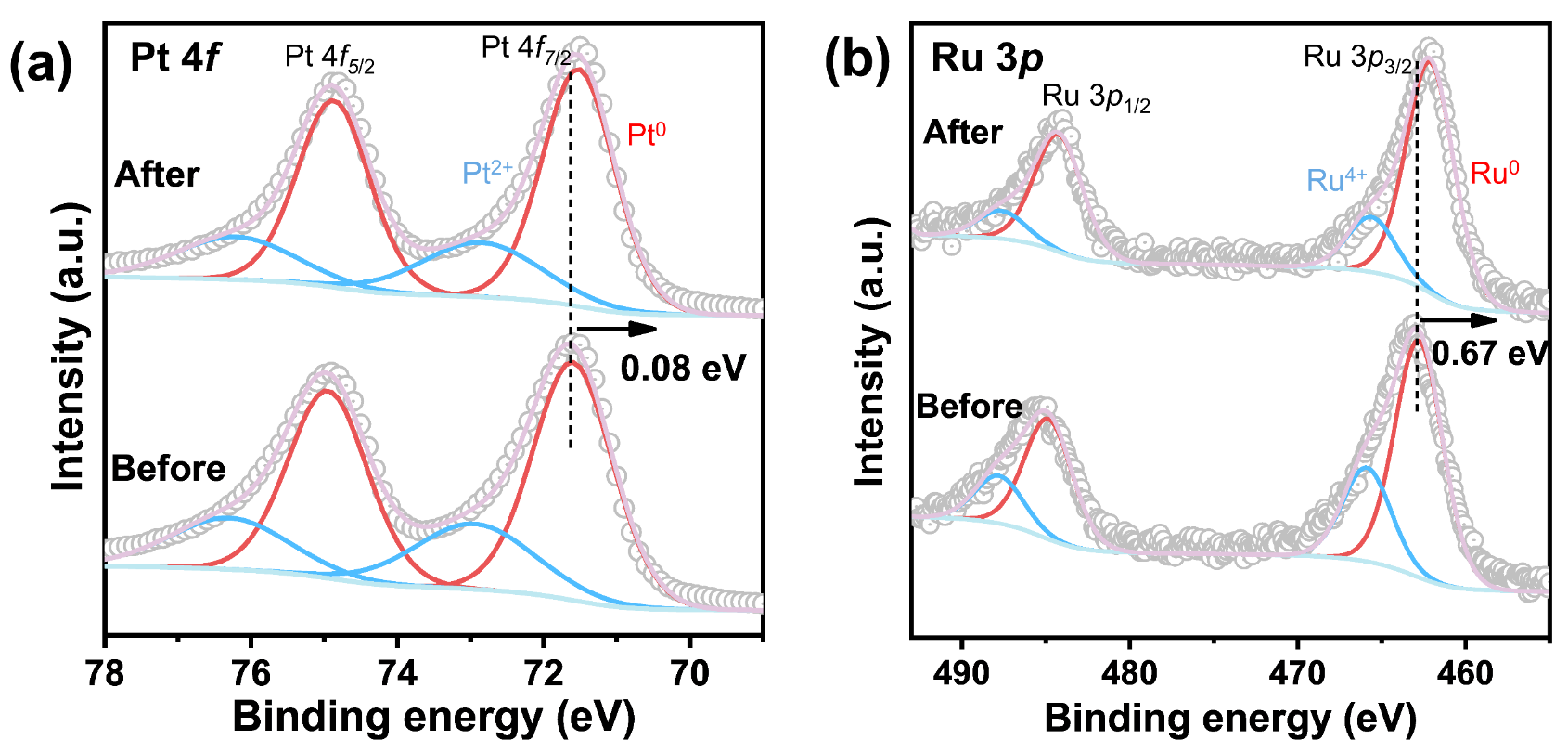


**Figure S31.** (a) Pt 4*f* and (b) Ru 3*p* XPS spectra of m-Pt-Ru after stability test.


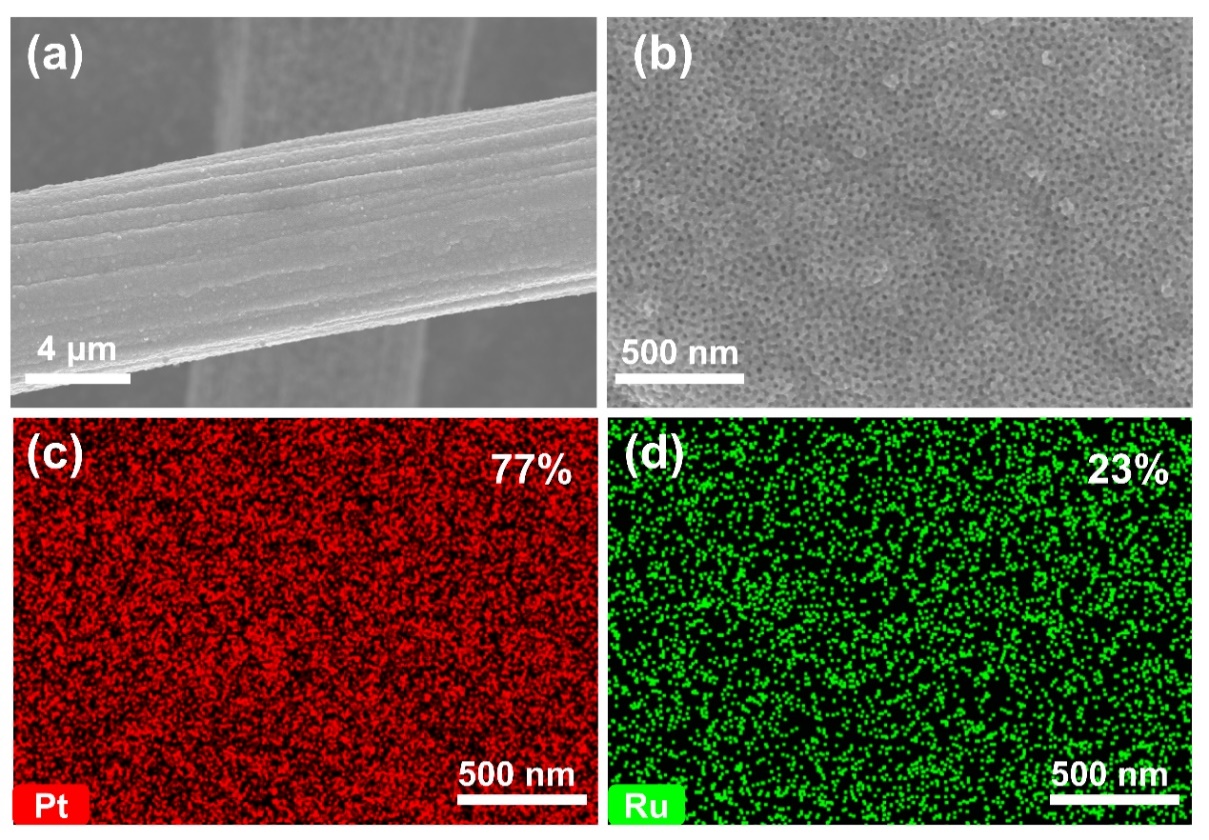


**Figure S32.** (a, b) SEM images and (c, d) EDS mapping images of m-Pt-Ru on carbon paper.


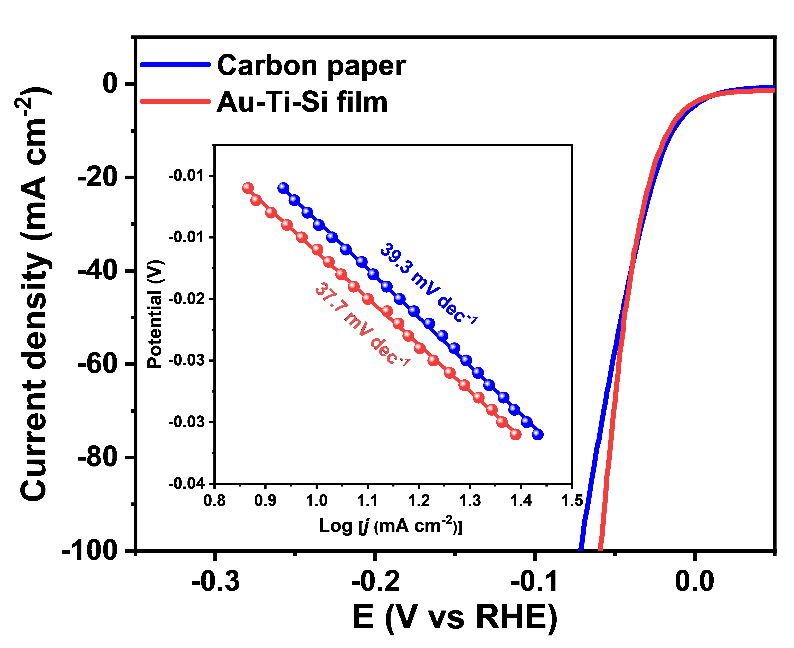


**Figure S33.** LSV curves of m-Pt-Ru on Au-Ti-Si substrate and carbon paper.


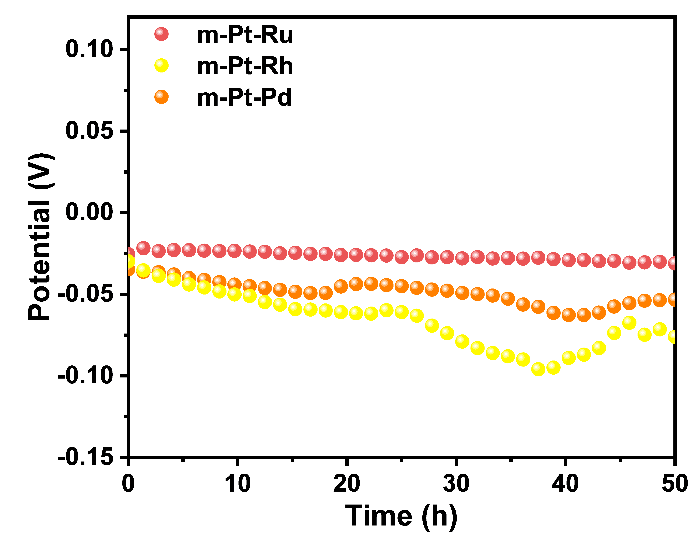


**Figure S34**. Chronopotentiometry curves of m-Pt-Ru, m-Pt-Rh, and m-Pt-Pd films.


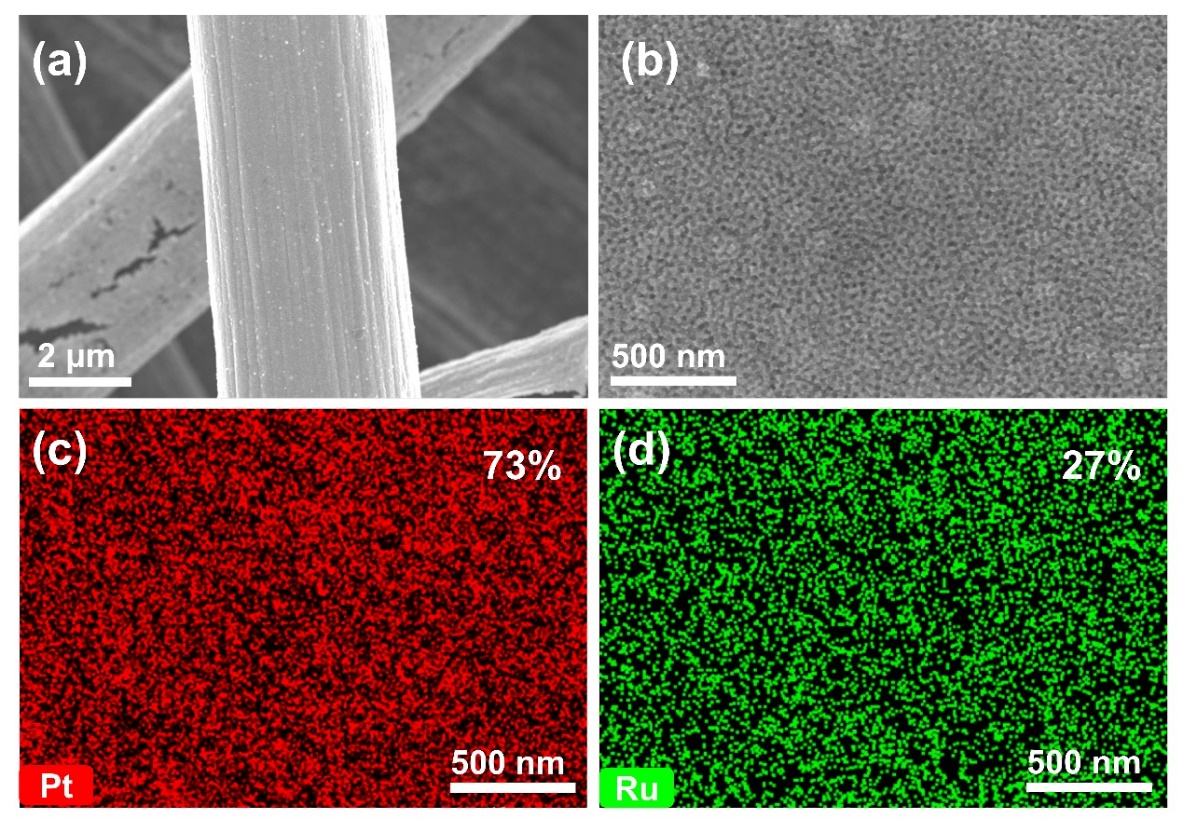


**Figure S35.** (a, b) SEM images and (c, d) EDS mapping images of m-Pt-Ru on carbon paper after stability test.


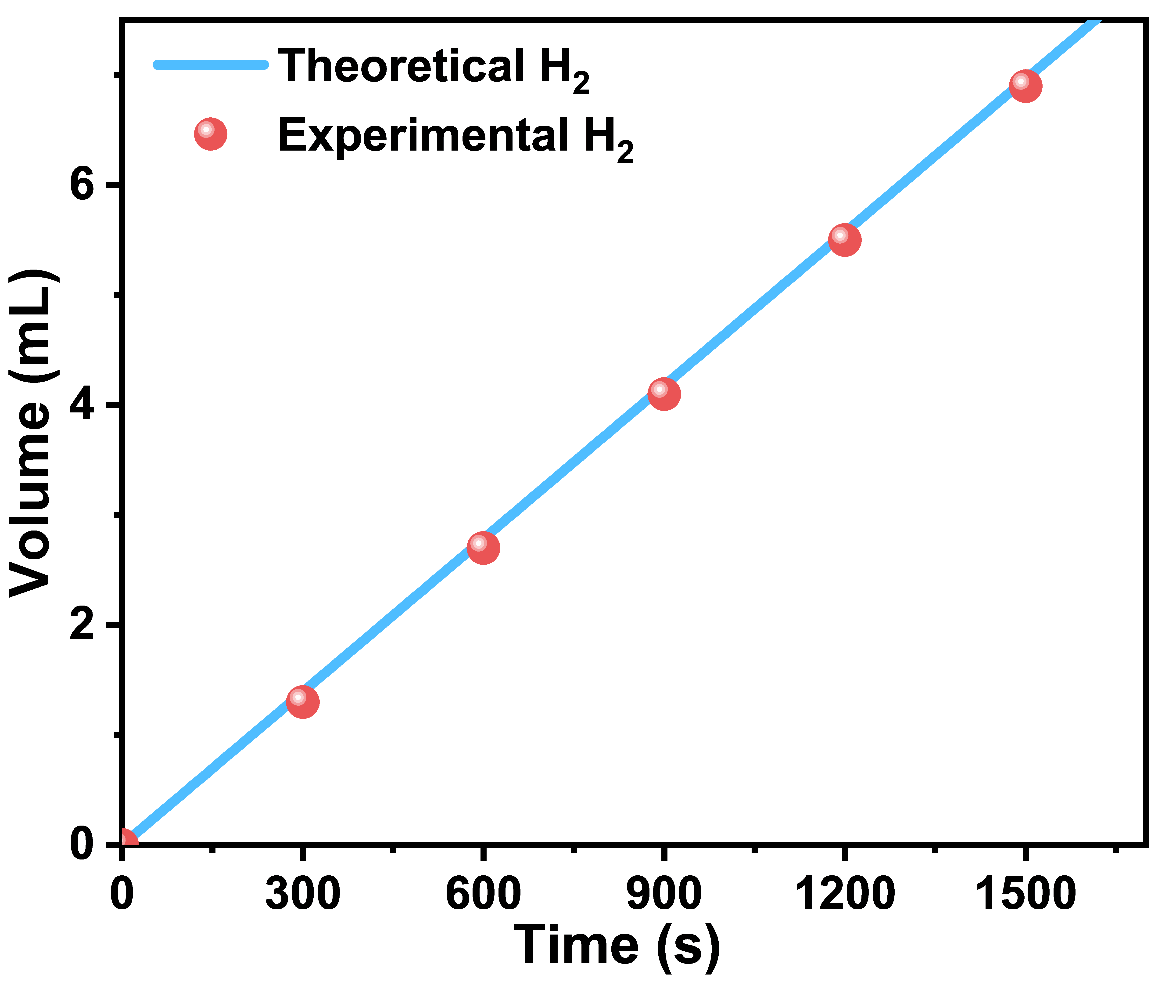


**Figure S36.** Experimental and theoretical amounts of hydrogen generated by the m-Pt-Ru at a current of 40 mA.

**Table S1.** Mass loadings of m-Pt and m-Pt-Ru measured by ICP-OES and the corresponding atomic ratios.

| **Samples** | **Pt**  **(mg)** | **Ru**  **(mg)** | **Pt**  **(atomic ratio%)** | **Ru**  **(atomic ratio%)** |
| --- | --- | --- | --- | --- |
| m-Pt | 0.066 | - | 100 | - |
| m-Pt-Ru | 0.027 | 0.004 | 78 | 22 |

**Table S2.** Fitting results of EIS spectra for nonporous Pt-Ru film and mesoporous Pt-Ru films with different pore sizes at an overpotential of 30 mV.

| Sample | R_1_ (Ω) | R_2_+R_3_ (Ω) |
| --- | --- | --- |
| Nonporous | 4.78 | 10.7 |
| 18 nm | 4.93 | 9.55 |
| 26 nm | 4.63 | 9.09 |
| 41 nm | 4.92 | 9.60 |

**Table S3**. Fitting results of EIS spectra for m-Pt-Au, m-Pt, m-Pt-Pd, m-Pt-Rh, and m-Pt-Ru at an overpotential of 20 mV.

| Sample | R_1_ (Ω) | R_2_+R_3_ (Ω) |
| --- | --- | --- |
| m-Pt | 4.74 | 23.5 |
| m-Pt-Rh | 4.78 | 15.1 |
| m-Pt-Ru | 4.64 | 14.1 |
| m-Pt-Pd | 5.29 | 17.9 |

**Table S4.** Summary of some recently reported Pt-based HER electrocatalysts.

| **Catalysts** | **η**  **(mV@10 mA cm^-2^)** | **Tafel slope**  **(mV dec^-1^)** | **Ref** |
| --- | --- | --- | --- |
| **m-Pt-Ru** | **16** | **37.7** | **This work** |
| FeCoNiCrPt HEA film | 18 | 21 | Energy Environ. Sci., 2024,17, 5854-5865 |
| D-NiO-Pt | 20 | 31.1 | Adv. Energy Mater., 2022, 12, 2200434 |
| Turing Pt | 25.6 | 28.2 | J. Am. Chem. Soc., 2024, 146, 8, 5355–5365 |
| CoPt-Pt_SA_ | 31 | 43.65 | Adv. Funct. Mater., 2022, 32, 2205920 |
| PdPtCuNiP | 32 | 37.4 | Adv. Funct. Mater., 2021, 31, 2101586 |
| Pt/MXene | 34 | 29.7 | Adv. Funct. Mater., 2022, 32, 2110910 |
| PtSi | 38 | 81 | Adv. Energy Mater., 2022, 12, 2200293 |
| PtSe_2_/Pt | 42 | 53 | Angew. Chem. Int. Ed., 2021, 60, 23388-23393 |
| FeCoPdIrPt@GO | 42 | 82 | Nat. Commun., 2020, 11, 2016 |
| Pt_1_/N-C | 46 | 36.8 | Nat. Commun., 2020, 11, 1029 |
| Pt@LDH | 58 | 43.6 | Small, 2023, 19, 2207044 |
| Pt–V_2_CT_x_ | 68.1 | 98.6 | Appl. Catal. B., 2022, 304, 120989 |
| Pt-NiCo LDO | 92 | 73 | Adv. Funct. Mater., 2024, 34, 2405919 |

**Table S5:** Calculated Bader charges of Pt and Ru atoms in binary Pt-Ru alloy, where surface atoms are highlighted in bold font.

| No. atom | Species | Total charge (*e*) | Gaining (+)/losing (–) charge (*e*) |
| --- | --- | --- | --- |
| 1 | Ru1 | 7.768775 | -0.231225 |
| 2 | Ru2 | 7.766952 | -0.233048 |
| 3 | Ru3 | 7.730145 | -0.269855 |
| 4 | Ru4 | 7.556491 | -0.443509 |
| 5 | Ru5 | 7.619548 | -0.380452 |
| 6 | Ru6 | 7.692692 | -0.307308 |
| 7 | Ru7 | 7.586801 | -0.413199 |
| 8 | Ru8 | 7.66593 | -0.33407 |
| 9 | Ru9 | 7.580872 | -0.419128 |
| 10 | Ru10 | 7.572908 | -0.427092 |
| **11** | **Ru11** | **7.71228** | **-0.28772** |
| **12** | **Ru12** | **7.759498** | **-0.240502** |
| **13** | **Ru13** | **7.690448** | **-0.309552** |
| **14** | **Ru14** | **7.726553** | **-0.273447** |
| 15 | Pt1 | 10.101292 | 0.101292 |
| 16 | Pt2 | 10.130953 | 0.130953 |
| 17 | Pt3 | 10.14721 | 0.14721 |
| 18 | Pt4 | 10.094148 | 0.094148 |
| 19 | Pt5 | 10.19102 | 0.19102 |
| 20 | Pt6 | 10.14153 | 0.14153 |
| 21 | Pt7 | 10.163319 | 0.163319 |
| 22 | Pt8 | 10.16088 | 0.16088 |
| 23 | Pt9 | 10.071967 | 0.071967 |
| 24 | Pt10 | 10.112488 | 0.112488 |
| 25 | Pt11 | 10.07772 | 0.07772 |
| 26 | Pt12 | 10.061184 | 0.061184 |
| 27 | Pt13 | 10.096572 | 0.096572 |
| 28 | Pt14 | 10.137115 | 0.137115 |
| 29 | Pt15 | 10.090233 | 0.090233 |
| 30 | Pt16 | 10.018666 | 0.018666 |
| 31 | Pt17 | 10.055103 | 0.055103 |
| 32 | Pt18 | 10.094258 | 0.094258 |
| 33 | Pt19 | 10.04713 | 0.04713 |
| 34 | Pt20 | 10.012675 | 0.012675 |
| 35 | Pt21 | 10.040955 | 0.040955 |
| 36 | Pt22 | 10.008367 | 0.008367 |
| 37 | Pt23 | 10.037228 | 0.037228 |
| 38 | Pt24 | 10.047999 | 0.047999 |
| 39 | Pt25 | 10.036333 | 0.036333 |
| 40 | Pt26 | 10.041888 | 0.041888 |
| 41 | Pt27 | 10.142246 | 0.142246 |
| 42 | Pt28 | 10.069455 | 0.069455 |
| 43 | Pt29 | 10.102423 | 0.102423 |
| 44 | Pt30 | 10.131445 | 0.131445 |
| 45 | Pt31 | 10.093272 | 0.093272 |
| 46 | Pt32 | 10.01039 | 0.01039 |
| 47 | Pt33 | 10.008138 | 0.008138 |
| 48 | Pt34 | 10.014369 | 0.014369 |
| 49 | Pt35 | 10.027516 | 0.027516 |
| 50 | Pt36 | 10.009682 | 0.009682 |
| 51 | Pt37 | 10.036423 | 0.036423 |
| 52 | Pt38 | 10.05008 | 0.05008 |
| **53** | **Pt39** | **10.119657** | **0.119657** |
| **54** | **Pt40** | **10.168356** | **0.168356** |
| **55** | **Pt41** | **10.197419** | **0.197419** |
| **56** | **Pt42** | **10.110985** | **0.110985** |
| **57** | **Pt43** | **10.136103** | **0.136103** |
| **58** | **Pt44** | **10.201642** | **0.201642** |
| **59** | **Pt45** | **10.125299** | **0.125299** |
| **60** | **Pt46** | **10.148505** | **0.148505** |
| **61** | **Pt47** | **10.132484** | **0.132484** |
| **62** | **Pt48** | **10.080189** | **0.080189** |
| **63** | **Pt49** | **10.108892** | **0.108892** |
| **64** | **Pt50** | **10.126901** | **0.126901** |
